# Unveiling Short‐Scale Responses: How Pico‐ and Nanoeukaryotic Plankton Navigate Environmental Variability in a Coastal Upwelling System

**DOI:** 10.1111/1758-2229.70070

**Published:** 2025-04-16

**Authors:** María Froján, Marta Muñoz‐Colmenero, Isabel G. Teixeira, Belén Arbones, Carmen G. Sotelo, Begoña Correa, Francisco G. Figueiras, Carmen G. Castro

**Affiliations:** ^1^ Instituto de Investigaciones Marinas (IIM), CSIC Vigo Spain; ^2^ Department of Genetics, Physiology and Microbiology Universidad Complutense de Madrid (UCM) Madrid Spain

**Keywords:** coastal upwelling, diversity, dynamics, metabarcoding, pico‐ and nanoeukaryotic plankton

## Abstract

For decades, identifying pico‐ and nanoeukaryotic plankton has been challenging due to their small size, leaving a significant gap in our knowledge of their composition and dynamics in comparison with their larger counterparts. The advent of molecular techniques unlocked new possibilities for exploring this hidden diversity. We applied metabarcoding targeting the V9 region of 18S rDNA to discern the principal taxonomic groups of pico‐ and nanoeukaryotes in the Ría de Vigo during the upwelling season. Nanoeukaryotes (NE) exhibit greater diversity compared to picoeukaryotes (PE). Specifically, NE were mainly comprised of nano‐sized diatoms and dinoflagellates, many of them uncategorized novel species. Within PE, *Syndiniales* and Marine Stramenopiles (MAST) were the main components identified. We also captured short‐term changes in the biomass and composition of PE an NE, with advection emerging as one of the most significant drivers. Most notably was the ocean inflow of unassigned picoeukaryotes into the Ría, likely driven by the negative circulation during downwelling. Moreover, local grazing within the Ría seems to be important enough to alter NE dynamics, but has a minimal effect over PE. Our findings improve the understanding of the small eukaryotic plankton community in coastal upwelling systems, highlighting a significant potential for novel diversity within these environments.

## Graphical abstract

We investigated the taxonomic composition and dynamics of pico‐ and nanoeukaryotes in an upwelling bay, providing insights into potential future trends. Advection and trophic interactions emerged as key factors shaping these communities. During downwelling, taxonomically unclassified picoeukaryotes and minor nanoeukaryotic taxa was dragged into the ria. Once upwelling restored, picoeukaryotes exited the ria, while no similar exit was observed for nanoeukaryotes, suggesting that local grazing may have influenced their dynamics.

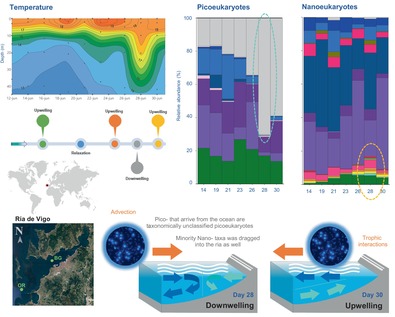

## 1 Introduction

Picoeukaryotes (PE, 0.2–2 μm) and nanoeukaryotes (NE, 2–20 μm) are key components of marine food webs and important drivers of global biogeochemical cycles (Massana 2011; Worden et al. 2015). They include ecologically diverse members such as primary producers, parasites, symbionts and predators/grazers that closely interact with each other and with bacteria and larger eukaryotes (Sherr et al. 2007; Massana and Pedrós‐Alió 2008; Bjorbækmo et al. 2020). Many of these tiny organisms are very similar in shape and unable to culture, so they are difficult to characterise by traditional microscopy or culture‐based methods (Massana 2011). Microscopy is time‐consuming and requires expert taxonomist, but provides key background data for evaluating new approaches. Cost‐efficient methods like HPLC pigment analysis and flow cytometry, provide information on pigment content and cell counts, but generally cannot resolve lower taxonomic levels (Vaulot et al. 2008). Overcoming the size limitation, recent advances in molecular biology, particularly 18S rDNA genomic techniques, are revolutionising our understanding of small eukaryotic plankton by revealing an extraordinary diversity in marine ecosystems (López‐García et al. 2001; Moon‐ van der Staay, De Wachter, and Vaulot 2001; Massana et al. 2004; Not et al. 2009; De Vargas et al. 2015; Hernández‐Ruiz et al. 2018; Obiol et al. 2020; Wang et al. 2021). A major challenge remains in connecting genetic biodiversity data to the functioning of the eukaryotic plankton community.

**FIGURE 1:**
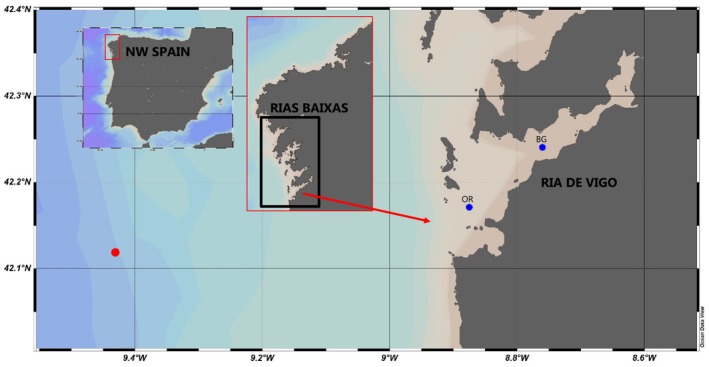
Location of the two sampling sites (BG and OR) in the Ría de Vigo, the southernmost of the Rías Baixas on Spain's northwest coast. The red dot denotes the position of the Silleiro buoy from ‘Puertos del Estado’.

Small plankton, such as PE and NE, are usually dominant in oligotrophic waters of the open ocean, whereas larger organisms, such as diatoms, typically thrive in coastal waters with high nutrient levels. However, there is an increasing recognition that small eukaryotes also play an important role in coastal and upwelling zones (Not et al. 2007; Collado‐Fabbri, Vaulot, and Ulloa 2011; Hernandez‐Ruiz et al. 2018; Gong et al. 2020). Indeed, it is well‐documented that specific oceanographic scenarios such as downwelling or upwelling relaxation lead to a dominance of smaller plankton in coastal upwelling waters off Chile (Iriarte and González 2004; Böttjer and Morales 2007) and NW Iberia (Lorenzo et al. 2005; Tilstone et al. 2003; Espinoza‐González et al. 2012), as well in some upwelling bays (Garrison 1976; Froján et al. 2014; Dames et al. 2023). An example of upwelling bays are the Rías Baixas (Figure 1), four V‐shaped coastal embayments, narrower and shallower inward, located at the northern limit of the Canary Current Upwelling System (Barton et al. 1998; Arístegui et al. 2009; Largier 2020). In this area, upwelling and downwelling occur year‐round, with upwelling most prominent in spring and summer, while downwelling prevails the rest of the year (Blanton et al. 1984). These upwelling and downwelling processes greatly influence the circulation of the ria and the exchange between the ria and the adjacent shelf (Barton et al. 2015). Upwelling leads to positive estuarine‐like circulation and promotes the ascent of nutrient‐rich subsurface water, which supports the growth of larger phytoplankton, particularly diatoms, enhancing primary production (Figueiras, Labarta, and Fernández‐Reiriz 2002; Figueiras et al. 2020). Positive circulation also promotes the offshore export of phytoplankton and dissolved organic carbon (Álvarez‐Salgado et al. 2003; Crespo et al. 2006). By contrast, several studies reported lower primary production during downwelling season, when the circulation pattern is reversed and small plankton (pico‐ and nanoplankton) dominate the microbial plankton community (Cermeño et al. 2006; Arbones et al. 2008; Froján et al. 2018). Since Margalef et al. (1955), significant knowledge has been gathered about microplankton in this area, primarily large diatoms and dinoflagellates. Species succession is well‐established and recognised to be intrinsically related to oceanographic variability (Figueiras, Labarta, and Fernández‐Reiriz 2002). Evidence also shows that upwelling promotes the proliferation of harmful species on the adjacent shelf which are then accumulated into the ria during downwelling (Fraga et al. 1988; Tilstone, Figueiras, and Fraga 1994; Fermín et al. 1996; Crespo and Figueiras 2007; Crespo et al. 2008). However, pico‐ and nanoplankton composition and dynamics have received much less attention, and it is only in recent times, thanks to the progress in molecular techniques, that it has become the focus of a more detailed study.

Our study investigates the biomass, diversity and taxonomic composition of PE and NE in the Ría de Vigo during the upwelling season, using both conventional and molecular methods. We hypothesize that biomass and composition of small eukaryotic assemblages are highly dynamic, responding to short‐term hydrographic changes. Understanding the drivers of this variability will provide insights on the microbial loop's functioning in upwelling regions, that is crucial to anticipate and manage the effects of global change in these highly productive ecosystems.

## 2 Experimental Procedures

### 2.1 Sampling Strategy

During the i‐SMALL‐1 cruise (June 12–30, 2017) in the Ría de Vigo on board the R/V *Mytilus*, samples were collected three times a week at two stations: BG (42°14.450′ N, 8°45.6180′ W) at the centre of the ria, and OR (42°10.2924′ N, 8°52.5222′ W) at the southern mouth (Figure 1). A CTD Sea‐Bird equipped with a fluorimeter and a rosette with 12 Niskin bottles were used to sample at five depths (surface, 10, 15, 20 and 40 m). Subsamples were taken to measure nitrate and size‐fractioned chlorophyll *a* (chl *a*) concentrations and size‐fractionated carbon fixation (CF). Two sampling depths at BG (surface and subsurface chl *a* maximum) were selected to evaluate the biomass and taxonomic composition of PE (0.2–2 μm) and NE (2–20 μm). Additionally, four surface samples from OR were selected during downwelling and the surrounding timeframe (June 23–30) to examine the composition of organisms transported from the shelf and exported from the ria (Table S1).

### 2.2 Upwelling Index and Current Velocities

Hourly wind speed and direction data from the Silleiro buoy (Figure 1) deployed by the Spanish Agency ‘Puertos del Estado’ were used to calculate Ekman transport perpendicular to the coast (*Q*_*x*_; m^3^ s^−1^ km^−1^) according to Bakun (1973):

$$-Q_{x}=-\frac{\rho_{a}*C_{d}*\left|V\right|*V_{y}}{\rho_{w}*f.}$$

where $\rho_{a}$ is the air density (1.22 kg m^−3^), $C_{d}$ is an empirical drag coefficient (1.3 × 10^−3^, dimensionless), $\left|V\right|$ is the wind speed (m s^−1^) and $V_{y}$ is the northern component of wind speed, $\rho_{w}$ is the density of seawater (1025 kg m^−3^) and f is the Coriolis parameter at this latitude (9.69*10^−5^ s^−1^). Positive values of −𝑄_𝑥_ indicate the predominance of northerly winds responsible for coastal upwelling, while negative values are related to downwelling processes due to southerly winds.

Current velocity profiles during the sampling period were determined with an Acoustic Doppler Current Profiler (ADCP—RDI 600 kHz) anchored to the seafloor at BG station (42 m depth). Another ADCP, an RDI 300 hHz, was moored at OR station (50 m depth). The measurement interval was 5 min for both ADCPs. Subtidal currents were extracted using a low‐pass symmetric digital filter (PL33) with a cut‐off period of 33 h (Beardsley, Limeburner, and Rosenfeld 1985) to remove tidal frequencies. This analysis was carried out in MATLAB.

### 2.3 Nitrate

Nitrate concentrations (μmol kg^−1^) were determined by segmented flow analysis using a Future‐Alliance autoanalyser following Hansen and Grasshoff (1983). The estimated analytical error was ±0.05 μmol kg^−1^.

### 2.4 Total and Size‐Fractioned Chlorophyll *a* and CF

For size‐fractionated chl *a* measurements 250 mL of seawater were sequentially filtered through 20, 2 and 0.2 μm pore‐size polycarbonate filters. The filters were stored at −20°C for at least 24 h. Pigments extraction involved immersing the filters in 30 mL of 90% acetone for 24 h in the dark at 4°C. Fluorescence of the extract was measured using a Turner Designs fluorimeter calibrated with pure chl *a* (Sigma Chemical Company). Chl *a* concentration (mg m^−3^) was calculated using Jeffrey and Humphrey (1975) equation. Total chl *a* concentration was the sum of the three size fractions: pico‐ (chl *a* < 2 μ), nano‐ (chl *a* 2–20 μm) and microphytoplankton (chl *a* > 20 μm).

Size‐fractionated CF was assessed by ^14^C uptake. At each sampling depth, three clear and two dark Corning bottles (75 mL) were filled with unfiltered seawater to which 185 kBq (5 μCi) of Na H^14^CO_3_ was added. Bottles were then placed into an isothermal chamber set to 15°C, with fluorescent lighting to simulate in situ conditions, which provided an average PAR of 240 μE m^−2^ s^−^1. The incubations lasted 2 h. Afterward, the content of each bottle was sequentially filtered through 20, 2 and 0.2 μm pore‐size polycarbonate filters. Subsequently, filters were exposed to HCl fumes for 12 h to remove unincorporated inorganic ^14^C and were then placed in vials with 4 mL of scintillation liquid. Disintegrations per minute (DPM) were measured in a liquid scintillation counter, using an external standard and the channel ratio methods to correct for quenching. Finally, the DPM measured in the dark bottles were subtracted from the DPM measured in the clear bottles to obtain the CF rates (μmol kg^−1^ h^−1^). These determinations, derived from short incubations, should not be interpreted as estimates of the system's net primary production. Instead, they were used to assess the importance of each size fraction in CF.

### 2.5 Pico‐ and Nanoeukaryotic Biomass

To determine the biomass of autotrophic picoeukaryotes (APE), 1.8 mL of seawater were collected in sterile cryovials and fixed with P + G solution (1% paraformaldehyde +0.05% glutaraldehyde) to a final concentration of 10%. Samples were then frozen in liquid nitrogen and stored at −80°C until analysed with a FACScalibur flow cytometer, using 0.6 mL aliquots. Each sample received 10 μL of yellow‐green fluorescent latex beads (Polyscience) as an internal standard (Calvo‐Díaz and Morán 2006). APE abundances were obtained, and their biomass was estimated according to Verity et al. (1992). As complementary information, the biomass of *Synechococcus* was quantified using the same procedure, following Lee and Fuhrman (1987). This group accounted for a small percentage (10% ± 8%) of the autotrophic picoplankton biomass during this study (Figure S1).

To determine the biomass of autotrophic nanoeukaryotes (ANE), heterotrophic nanoeukaryotes (HNE) and heterotrophic picoeukaryotes (HPE), 10 mL of seawater were fixed with a buffered solution of formaldehyde (2% final concentration) and stained with DAPI (0.1 μm ml^−1^ final concentration) for 10 min in the dark (Porter and Feig 1980). Samples were then filtered through 0.2 μm Milipore‐Isopore black filters and were frozen at −20°C until analysis with an epifluorescence microscope at ×1000 magnification. Under blue light, autotrophic organisms were identified and counted by their red colour. Under UV light, due to DAPI staining of nucleic acids, all organisms were counted. Heterotrophic organisms were obtained by subtracting the counts of autotrophic organisms from the total organisms counted. In each sample, at least 300 cells were counted. Biovolumes were calculated by measuring the diameter of several individuals (at least 25 individuals in each group and sample) assuming a spherical shape. The carbon biomass was estimated according Verity et al. (1992) for PE and NE.

### 2.6 DNA Extraction, Amplification and Sequencing

Water samples were sequentially filtered through 20, 2 and 0.2 μm pore‐size polycarbonate filters (Filter Lab, Filtros Anoia, S.A.), separating the three size fractions, microplankton, nanoplankton and picoplankton, respectively. Pico‐ and nanoplankton filters were used for the DNA extraction. This extraction was performed with the commercial kit DNeasy Power Water Kit (QIAGEN), following manufacturer's instructions. The extracted DNA was quantified (ng μL^−1^) by fluorometry, using a Qubit v3.0 fluorimeter (Life Technologies) and a Qubit dsDNA HS assay kit (Qubit; ref.: Q32851), and the DNA quality was also examined with a spectrophotometer through the ratio A260/A280. The DNA extracted was amplified by polymerase chain reaction (PCR) using the commercial polymerase kit ‘Ready to go’ (GE Healthcare; ref.: 407513‐SRT), following the manufacturer's conditions. Primers designed by Amaral‐Zettler et al. (2009) were used to amplify a fragment of the hypervariable V9 region of the 18S rRNA gene in eukaryotes with a variable amplicon size of 87–186 bp. These primers can also amplify some non‐specific 16S rRNA in prokaryotes. The polymerase chain reaction (PCR) conditions were an initial cycle at 95°C for 3 min, followed by 30 cycles of 30 s at 95°C, 30 s at 57°C for the annealing of the primers and 1 min at 72°C for elongation, plus 7 min at 72°C for extra elongation. This PCR was run in a Veriti Thermal Cycler (Applied Biosystems) and positive amplifications were visualised by agarose gel electrophoresis with RedSafe staining (×20,000, Intron Biotechnology). Negative controls showed no amplification.

The PCR products were purified using the ‘AMPure XP’ reagent (Beckman Coulter)/‘MAG‐BIND Total Pure NGS’ (OMEGA), and all PCR products were quantified again by fluorimetry using Qubit 3.0. The samples were barcoded with ‘Ion Express barcode adapters 1–16’ (Thermo Fisher) using the ‘Ion Plus Fragment Library kit’ (Thermo Fisher) for binding these barcodes and adapters for the Ion Personal Genome Machine sequencer (PGM) and preparing the amplicon libraries. The purification of these libraries was carried out using again ‘AMPure XP’ reagent and the libraries were double quantified by Qubit 3.0 and qPCR, with the ‘Ion Library TaqMan Quantification Kit’ (Thermo Fisher) in an ABI 7500 Fast Real‐time PCR system. Additionally, the quality of the prepared libraries was determined with an Agilent 2100 Bioanalyzer. Six equimolar pools were prepared with a concentration of 6.23 pM the first two of them and 8 pM the other four, and they were loaded in two Ion 314R Chip v2 BC and four 316 Chip v2 (Life Technologies), respectively, using the Ion PGM Hi‐Q Chef (Life Technologies) with the proper reagents for this machine. The loaded chips were sequenced on an Ion PGM using 500 flows. PGM software filtered low‐quality or polyclonal sequences, trimmed adapters and barcodes, giving one demultiplexed file per sample (.fastq and .bam formats).

### 2.7 Sequencing Data Quality and Bioinformatics Analyses

All sequenced samples were quality‐filtered using a Q20 as threshold and minimum length of 80 bp with Cutadapt program v1.16 (Martin 2011). Filtered sequences were imported to QIIME v1.9 (Caporaso et al. 2010) as it was recommended in Muñoz‐Colmenero et al. (2021) for this type of samples sequenced with PGM and were clustered into Operational Taxonomic Units (OTU) performed with 99% similarity and testing both directions. The script used was pick_open_reference_otus.py. Taxonomy was assigned UCLUST program (Edgar 2010) by default. The taxonomy was assigned using 99% of identity and SILVA 132 database. Once the taxonomy was assigned, those taxa with less than 10 reads in the dataset were removed. All samples were normalised to the same number of reads by rarefaction to make all samples comparable to each other.

Bacteria and Archaea taxa were removed from these analyses using the script filter_taxa_from_otu_table.py, as well as, large protist, Metazoan and Fungi, since the objective was focused into the study of PE and NE. The dataset was separated by filter (PE vs. NE) in order to analyse the communities separately. Additionally, during the preparation of the OTU table for further analysis we removed the unknown non‐eukaryotes OTUs classified as ‘unassigned’ which represent the 28% of PE and 20% of NE reads. We also meticulously identified and excluded OTUs at the genus level, that according to the literature, clearly exceed the size thresholds for PE and NE, which represented a very small proportion. Following this, samples were imported into QIIME2 (Bolyen et al. 2019) to make some analysis and calculations. For BG samples, the alpha diversity (observed_otus, Chao1, Simpson and Shannon) was calculated for each filter and significance was tested using the Mann–Whitney test. The plots were performed using R studio software. PCoA ordination plot was calculated, based on Bray Curtis metrics and the plots were drawn with R studio. ANOSIM test was applied to confirm the plankton community recovered with each filter was different.

For each size fraction separately, the variation of the plankton community along the sampled period was studied. Barplots of taxa abundances were drawn for each station (BG and OR) using SigmaPlot. PCoA (QIIME2) and UPGMA (QIIME v1.9) were used to assess sample similarity. To check if the samples could be grouped into two clusters according to the moment of sampling, before or after the downwelling event, an ANOSIM test was used. Kruskal–Wallis test, non‐parametric *t* test and LEfSe analysis (Segata et al. 2011) were used to know what taxa were recovered with significantly different read relative abundance according to the downwelling event (before or after). Principal component analysis (PCA) was plotted with IBM SPSS to test trends in the relative abundance of the most relevant taxa of PE and NE identified in this study. A SIMPER analysis was perform using the PAST software to identify the taxa contributing most to the observed variability. Additionally, a PCA was conducted in PAST, incorporating explanatory variables, to assess the main abiotic drivers of variability in the pico‐ and nanoeukaryotic plankton community.

## 3 Results

### 3.1 Wind Forcing and Oceanographic Setting

**FIGURE 2:**
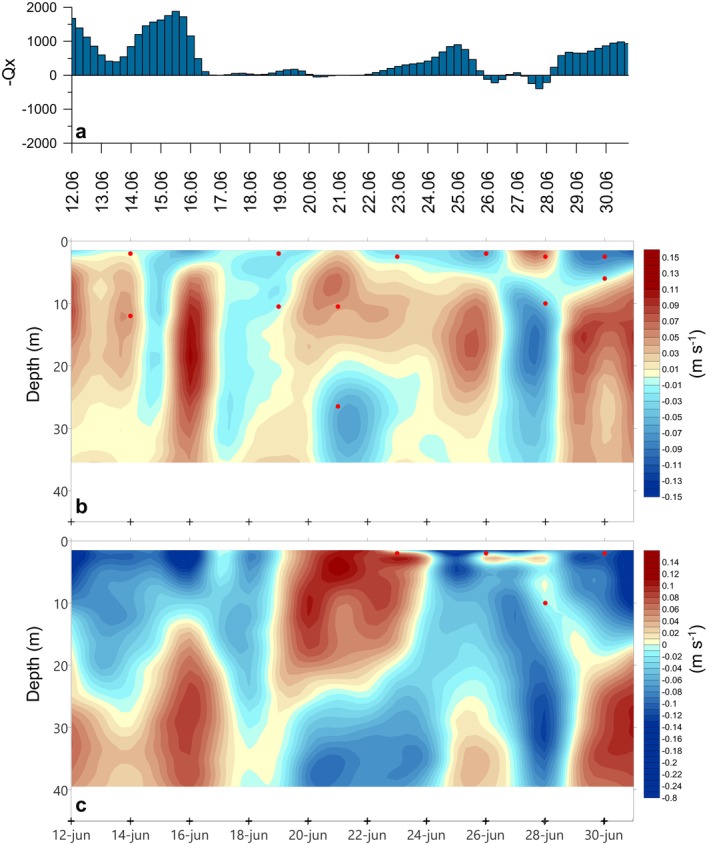
(a) Time series of upwelling index (*Q*_*x*_, m^3^ s^−1^ km^−1^). Positive values indicate upwelling and negative values indicate downwelling. Subtidal current velocities (m s^−1^) parallel to the main axis of the Ría de Vigo at (b) BG and (c) OR stations. Positive values (red) indicate inflow velocities and negative values (blue) indicate outflow velocities respect to the ria. Discrete sampling depths for Dapi and molecular analysis are indicated by red dots.

**FIGURE 3:**
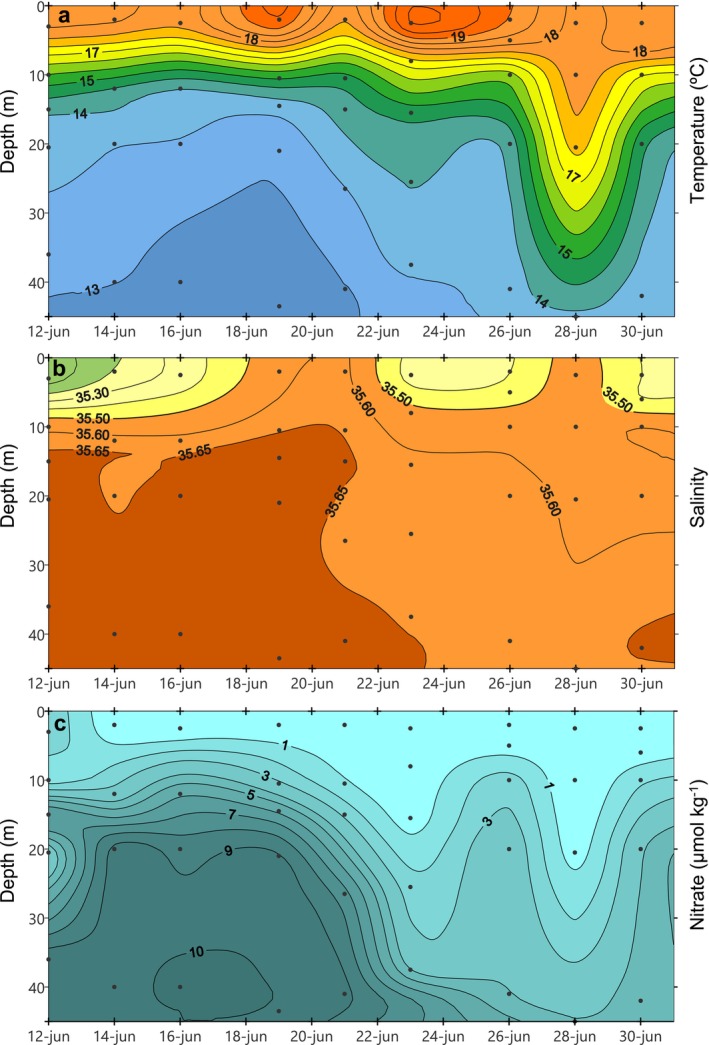
(a) Temperature (C), (b) salinity and (c) nitrate concentration (μmol kg^−1^), at BG station.

We found five short‐term hydrographic scenarios over 3 weeks of semi‐intensive sampling during the upwelling season. Using the upwelling index, as well as the circulation pattern of the ria, we were able to distinguish a succession of upwelling‐relaxation‐upwelling‐downwelling‐upwelling (Figures 2 and 3). The beginning of the sampling (June 12–16) coincided with upwelling‐favourable winds (−*Q*_*x*_ positive values, Figure 2a) which reinforced the positive estuarine circulation of the ria, with water inflow at depth and outflow at surface, observable both at BG (Figure 2b) and OR (Figure 2c) stations. This circulation decreased surface salinity (< 35.50), lowered the temperature (< 13°C) and nitrate enriched (> 10 μmol kg^−1^) the deep water layer (Figure 3). Afterwards, between June 17 and 23, a period of relaxation was recorded (Figure 2a) during which the circulation pattern was reversed, more evidently in OR (Figure 2c). This relaxation period was initially characterised by a strong thermal stratification, reaching up to 6°C of difference between sea surface and 30 m depth (Figure 3a). As the days progressed, thermal stratification weakened and nitrate levels decreased (< 1 μmol kg^−1^) above the thermocline (Figure 3c). This upwelling‐relaxation cycle was followed by another short upwelling event (June 24–25), which again induced positive estuarine circulation in the ria (Figure 2b,c) and it can be easily recognisable by the uplift of temperature and nitrate isolines (Figure 3a,c). Subsequently, on June 26–28 (Figure 2a) wind conditions reversed temporally (−*Q*_*x*_ negative values) and water entered the ria at surface and exited at depth (Figure 2b). This downwelling event warmed the whole water column, pushing the thermocline downwards (Figure 3a). Also, surface nitrate‐depleted water (< 1 μmol kg^−1^) was dragged up to 20 m depth. On the last days of sampling (June 29–30) upwelling and positive circulation was restored, bringing again cold and nutrient‐rich subsurface water to the surface (Figure 3).

### 3.2 Total and Size‐Fractionated Chlorophyll *a* and CF

**FIGURE 4:**
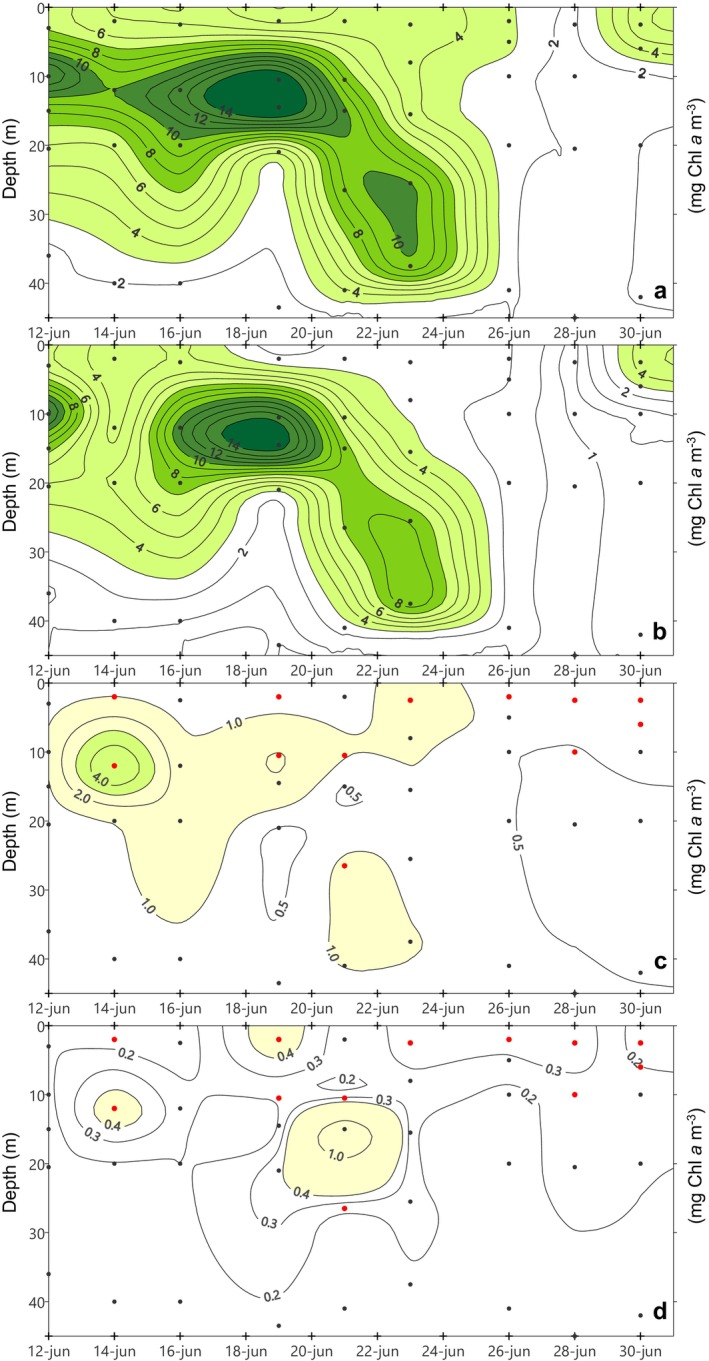
Total and size‐fractionated chl *a* (mg m^−3^) at BG station: (a) Total chl *a*, (b) chl a (> 20 μm), (c) chl *a* (2–20 μm), (d) chl *a* (< 2 μm). Red dots denote discrete sampling depths for Dapi and molecular analysis.

Throughout the period addressed, the observed total chlorophyll *a* concentration (chl *a*) at BG station showed a highly variable distribution directly connected with the temperature pattern (*r* = −0.81; *p* < 0.01). Depth‐integrated total chl *a* at BG station averaged 201 ± 97 mg m^−2^ over the whole sampling period (Table 1). Higher water column integrated values were recorded during upwelling and relaxation periods, while the lowest levels were found during the downwelling event (June 28). More specifically, during the initial upwelling‐relaxation cycle, a subsurface chl *a* maximum (14 mg m^−3^) developed at the thermocline depth, associated with the intense stratification (Figure 4a). From June 21 onwards, there was a gradual weakening of stratification leading to the sinking of chl *a*. After that, during downwelling (June 26–28), chl *a* concentration sharply declined, nearly vanished, forced by the negative circulation in the ria. Finally, chl *a* concentration showed a rising trend again at surface on June 29–30, responding to new nutrient inputs from the last upwelling. Short‐term variations of hydrographic conditions were also reflected in the size‐structure of the phytoplankton community. The pattern observed in the depth‐integrated total chl *a* was repeated by microphytoplankton size‐fraction (> 20 μm), and this size‐class made up the largest portion of total chl *a* (Table 1). Likewise, the central role of large phytoplankton during upwelling and its loss of prominence during downwelling becomes evident when comparing the different size‐class distributions in the water column (Figure 4b–d). Nanophytoplankton (2–20 μm), was the second in importance and the lowest contribution came from picophytoplankton (< 2 μm) (Table 1). However, both together, pico‐ and nanophytoplankton, accounted for well over half of total chl *a* on June 28, under downwelling conditions.

**TABLE 1:** Total and size‐fractionated chlorophyll *a* concentration (chl *a*, mg m^−2^) and carbon fixation (CF; mg C m^−2^ h^−1^) integrated over the entire water column at BG station, together with the percentage corresponding to each size‐fraction (in parenthesis). At the bottom of the table, average ± SD for the entire period (June 2017). Size fractionation: micro (> 20 μm), nano (2–20 μm) and pico (< 2 μm). Shaded data correspond to total chl *a* and total PP.

	Total chl *a*	Micro chl *a*	Nano chl *a*	Pico chl *a*	Total CF	Micro CF	Nano CF	Pico CF
12/06/2017	240	206 (86)	28 (12)	6 (3)	511	465 (91)	33 (6)	13 (3)
14/06/2017	228	143 (63)	75 (33)	9 (4)	310	273 (88)	29 (9)	8 (3)
16/06/2017	305	248 (81)	50 (16)	7 (2)	833	760 (91)	61 (7)	12 (1)
19/06/2017	263	213 (81)	37 (14)	12 (5)	312	273 (88)	34 (11)	5 (1)
21/06/2017	280	225 (80)	37 (13)	17 (6)	423	366 (86)	49 (12)	8 (2)
23/06/2017	265	215 (81)	39 (15)	10 (4)	353	302 (85)	44 (12)	8 (2)
26/06/2017	101	70 (69)	25 (24)	7 (6)	79	59 (75)	16 (21)	4 (5)
28/06/2017	53	24 (45)	19 (36)	10 (19)	80	32 (41)	32 (40)	15 (19)
30/06/2017	73	44 (61)	20 (28)	8 (11)	105	82 (78)	17 (16)	6 (6)
*Entire period*	201 ± 97	154 ± 87 (72 ± 14)	37 ± 18 (21 ± 9)	10 ± 4 (7 ± 5)	334 ± 243	290 ± 229 (80 ± 16)	35 ± 14 (15 ± 10)	9 ± 4 (5 ± 6)

**FIGURE 5:**
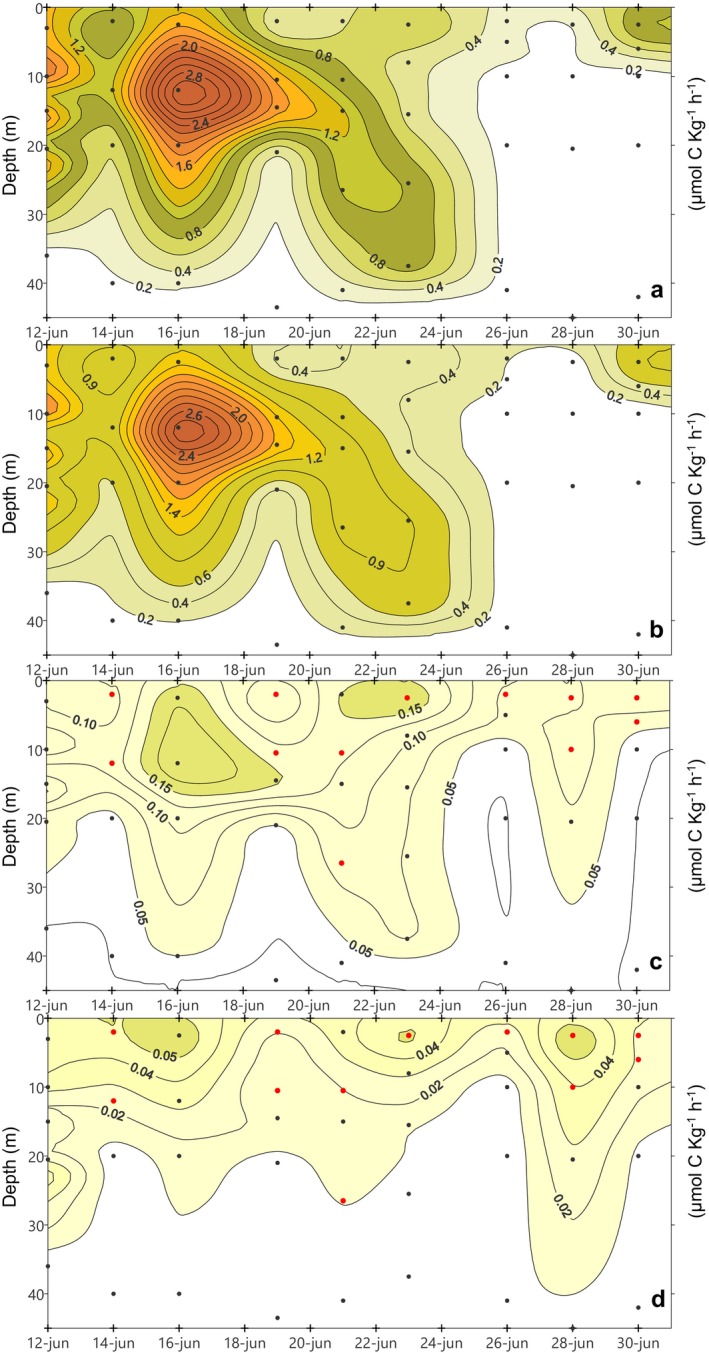
Total and size‐fractionated CF (μmol kg^−1^ h^−1^) at BG station: (a) Total CF, (b) PP (> 20 μm), (c) CF (2–20 μm) and (d) CF (< 2 μm). Discrete sampling depths for Dapi and molecular analysis are indicated by red dots.

The variability in depth‐integrated total CF at BG was large (334 ± 243 mg C m^−2^ h^−1^) with a chlorophyll‐like pattern (*r* = 0.83; *p* < 0.01) (Table 1). Also, the CF distribution in the water column (Figure 5a) closely resembled that of total chl *a* (Figure 4a). Upwelling/relaxation led to a subsurface CF maximum on June 16 (3 mg C kg^−1^ h^−1^), which sank a few days later. After that, CF levels were low under downwelling while recovery somewhat on the last day of the sampling at surface (Figure 5a). Microphytoplankton accounted for the largest fraction of total CF and repeated the vertical distribution of chl *a* > 20 μm, characterised by the sinking of its maximum driven by relaxation, to nearly disappear during downwelling (Figure 5b). Nanophytoplankton contribution to total CF was relatively small while that of picoplankton was even lower (Table 1). Only on June 28, under downwelling conditions, the joint contribution of the two smaller size‐fractions exceeded that of large phytoplankton. At this moment, vertical distribution of small (Figure 5c,d) and large (Figure 5b) phytoplankton differed. In particular, a downward flux of small cells was recorded, but unlike microphytoplankton, small phytoplankton did not vanish from water column when reversal circulation was established, quite the opposite, picophytoplankton CF peaked at surface.

### 3.3 The Biomass and Trophic Nature of PE and NE

Throughout the study period, the average total biomass of PE and NE in surface waters at BG station was comparable (Table S2) and their dynamics appeared to be related with different oceanographic scenarios (Figures 2 and 3), showing a parallel pattern of lower values during the relaxation (June 19–21) and maximum values during the downwelling event (June 28). Moreover, the total biomass was unequally distributed between autotrophs and heterotrophs (Table S2), with a marked predominance of APE and ANE over their heterotrophic counterparts (HPE and HNE). On the other hand, subsurface samplings from BG station replicate the aforementioned variability at surface, with both PE and NE increasing their biomass during downwelling (Table S2). While autotrophic components remained predominant, the increasing biomass ratios of HPE and HNE points to a higher proportion of heterotrophic organisms in subsurface waters, particularly evident during the relaxation on June 21 (Table S2).

**FIGURE 6:**
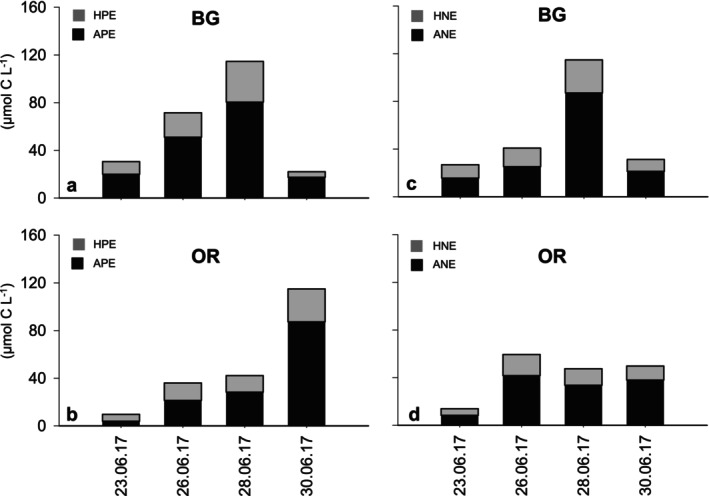
Pico‐ (PE) and nanoeukaryotes (NE) biomass at BG and OR stations at surface (μg C L^−1^): (a) APE and HPE at BG station, (b) APE and HPE at OR station, (c) ANE and HNE BG station and (d) ANE and HNE at OR station. APE (biomass of autotrophic picoeukaryotes), HPE (biomass of heterotrophic picoeukaryotes), ANE (biomass of autotrophic nanoeukaryotes) and HNE (biomass of heterotrophic nanoeukaryotes).

In the outermost location of the Ría de Vigo (OR), the biomass of PE at the surface exhibited a different pattern for the last 4 days of sampling compared to that of BG station (Figure 6a,b and Table S2). In fact, the biomass at these two locations showed no significant correlation (*r* ^2^ = 0.11; *p* > 0.05). Notably, OR experience a progressive increase in total PE biomass at the surface during downwelling, peaking on June 30 (115 μg C L^−1^) when upwelling conditions were restored (Figure 6b and Table S2). This rise was attributed to both APE and HPE (Figure 6b). This is rather interesting given that total PE biomass at BG peaked only 2 days earlier, on June 28 (115 μg C L^−1^) during downwelling conditions, but declined right after (Figure 6a). Another important factor to consider is that NE biomass also peaked at BG station on June 28 (Figure 6c), but did not increase at OR on the final day of sampling (Figure 6d), behaving differently to the smallest size fraction (Figure 6b).

### 3.4 Small Eukaryotic Plankton Diversity, Composition and Major Taxonomic Groups

**FIGURE 7:**
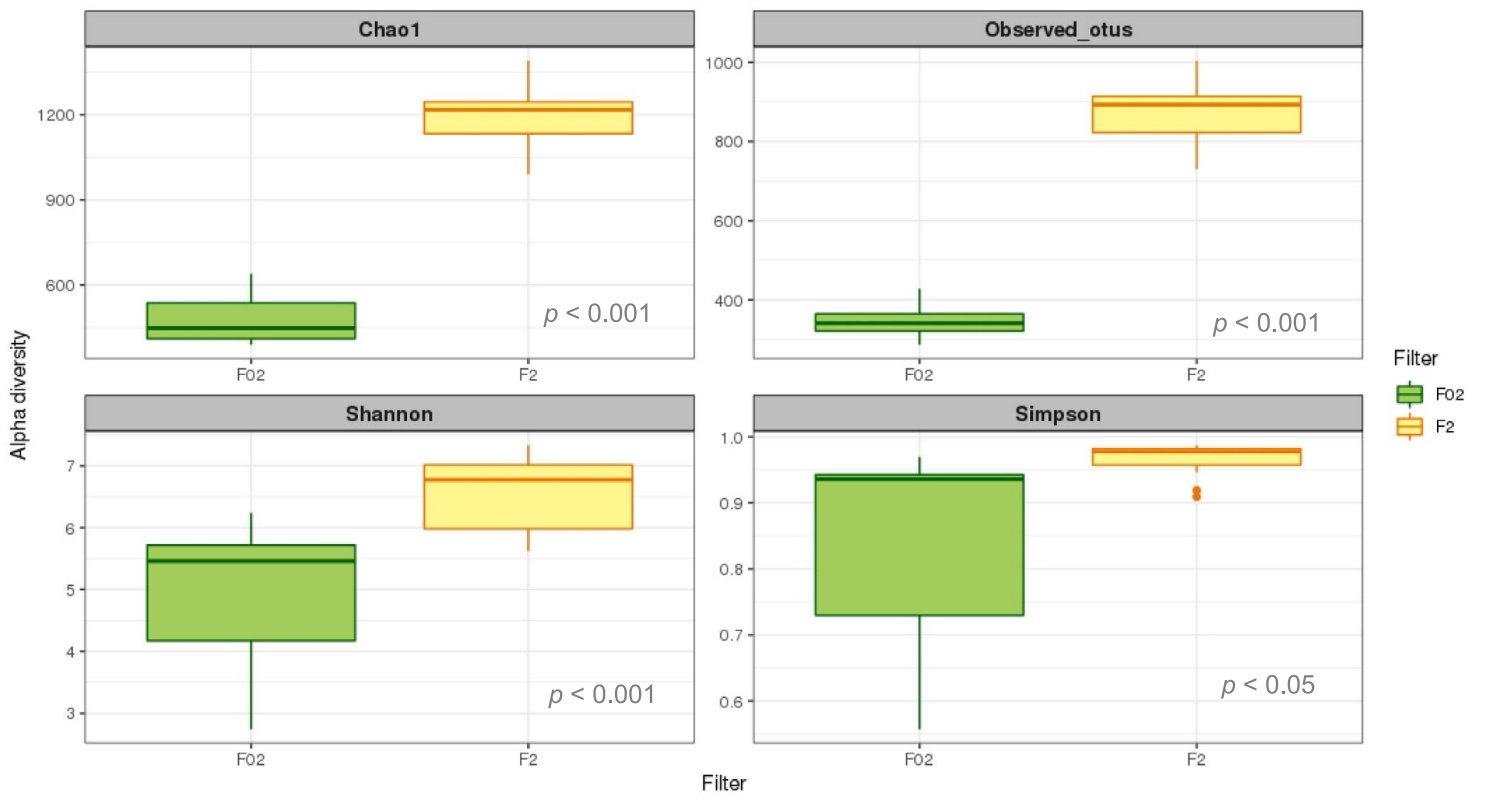
Box plots of Alpha diversity indices: Chao1 (a, Observed_otus (b), Shannon (c) and (1D) Simpson's index of diversity (d) for picoeukaryotes (PE in green) and nanoeukaryotes (NE in yellow) communities at BG station. Difference between PE and NE was tested using a Wilcoxon–Mann–Whitney test.

The June environmental DNA survey at BG station yielded an average of 240,344 reads per sample from 24 samples, which was reduced to 134,205 after quality checks. Sequence clustering identified 3648 different OTUs (excluding ‘unassigned’, large protist, Metazoan, Bacteria, Archaea and Fungi). The dataset was categorised by filter (PE: 0.2–2 μm; NE: 2–20 μm) to analyse the diversity and taxonomic composition of the PE and NE communities, separately. To test significant differences in alpha diversity between PE and NE, we also calculated Chao 1, Observed OTUs, Shannon and Simpson (1D) indices (Figure 7). All metrics indicated greater richness and diversity of NE compared with PE (*p* < 0.001).

**FIGURE 8:**
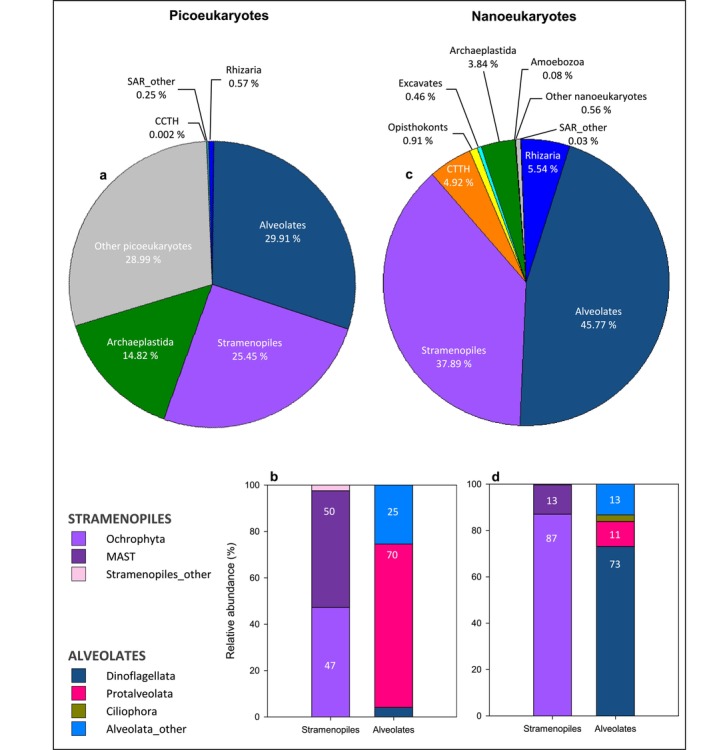
Overview of the taxonomic composition of (a) picoeukaryotes and (c) nanoeukaryotes at supergroup level detected in surface waters at BG station. Relative abundance (%) of the different components of Stramenopiles and Alveolates belonging to (b) picoeukaryotes and (d) nanoeukaryotes.

**FIGURE 9:**
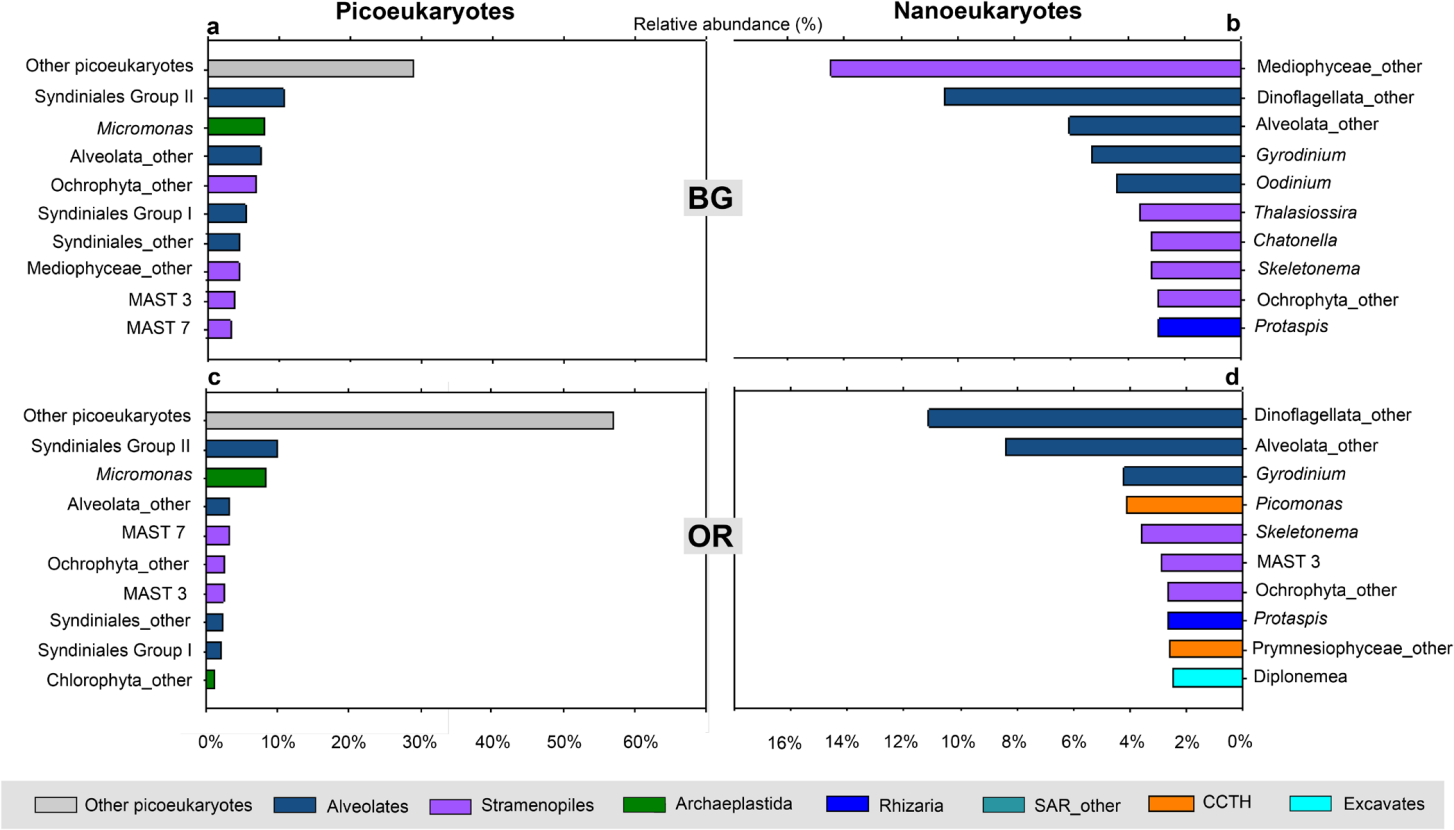
Top 10 most abundant taxa of: (a) picoeukaryotes and (b) nanoeukaryotes in surface waters at BG station; and (c) picoeukaryotes and (d) nanoeukaryotes in surface waters at OR station. Legends bellow report the different supergroup affiliation.

The phylogenetic diversity of picoeukaryotes at BG station at surface mainly involved sequences of the SAR (Stramenopiles, Alveolates, Rhizaria) and Archaeplastida supergroups (56 and 15%, respectively), as well as a high proportion of OTUS classified at the general level of ‘Eukaryotes’ (29%), which we designate as ‘other picoeukaryotes’ (Figure 8a). Within SAR supergroup, Stramenopiles (25%) and Alveolates (30%) had the highest abundances while Rhizaria represented < 1%. The main components of Stramenopiles were Ochrophyta and MAST (Marine Stramenopiles), which were found in nearly equal shares (Figure 8b). Alveolates, instead, were predominantly represented by Protalveolata (70%). Meanwhile, the supergroup Archaeplastida was entirely comprised by the Chloroplastida clade. At a deeper taxonomic rank, the top 10 most abundant groups of picoeukaryotes (Figure 9a) were led by the aforementioned unknown category ‘other picoeukaryotes’ (29%), followed by *Syndiniales* Group II (11%) and *Micromonas* (8%). Also, MAST 3 and MAST 7 accounted for 7% of the total read abundance.

On the other hand, SAR supergroup also accounted for the largest share of the phylogenetic diversity of nanoeukaryotes at BG station at surface (89%, Figure 8c), but in this case strongly represented by Alveolates (46%) and Stramenopiles (38%). The Stramenopiles group was mainly composed of Ochrophyta while Dinoflagellata was the most prominent member of Alveolates (Figure 8d). At lower taxonomic level (Figure 9b), dominant groups included centric diatoms, such as *Thalassiosira* (4%), *Skeletonema* (3%) and particularly an unclassified centric diatom ‘Mediophyceae_other’ (14%) at the top of the list. Moderate abundances were also observed in Dinoflagellata group, including Gyrodinium (5%), the parasite *Oodinium* (4%) and ‘Dinoflagellata_other’ (10%).

Below the surface layer at BG station, the taxonomic composition at the supergroup level (Figure S2) closely resembled that observed at surface (Figure 8). However, at higher taxonomic resolution, some dissimilarities emerged. For example, PE showed an increase in *Syndiniales* members at subsurface (Figure S3a), reaching up to 36% of the relative abundance if the whole *Syndiniales* group is considered, which means that almost doubled their relative abundance in surface waters (Figure 9a), and the same applies to MAST members. Additionally, *Syndiniales* Group I was also listed within the top 10 most abundant nanoeukaryotic taxa in subsurface waters (Figure S3b), whereas the unclassified ‘Mediophyceae_other’ declined in importance compared to surface (Figure 9b).

**FIGURE 10:**
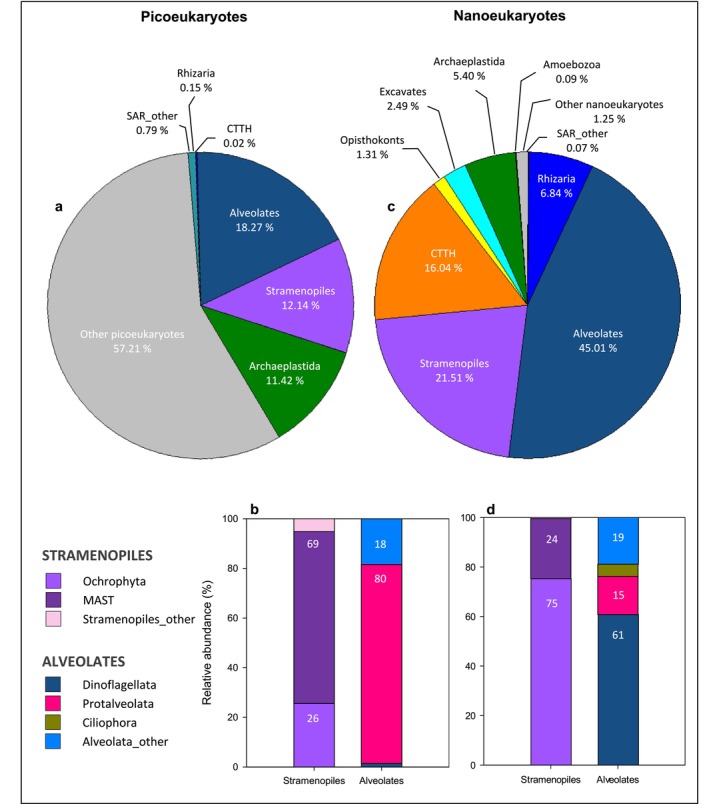
Overview of the taxonomic composition of (a) picoeukaryotes and (c) nanoeukaryotes at supergroup level detected in surface waters at OR station (only the last four sampling days). Relative abundance (%) of the different components of Stramenopiles and Alveolates belonging to (b) picoeukaryotes and (d) nanoeukaryotes.

The PE community in surface waters at OR station (Figure 10a) was largely dominated by ‘other picoeukaryotes’ (57%), while the proportion of Stramenopiles and Alveolates was about half of that at BG station. By contrast, less abundant NE at BG station, like CCTH and Excavates (Figure 8b), increased substantially at the outermost station OR (Figure 10b). Indeed, two CCTH members (*Picomonas* and Prynesiophyceae_other) and *Diplonemea* ranked among the 10 most abundant taxa at this location (Figure 9d).

### 3.5 Small Eukaryotic Plankton Dynamics

**FIGURE 11:**
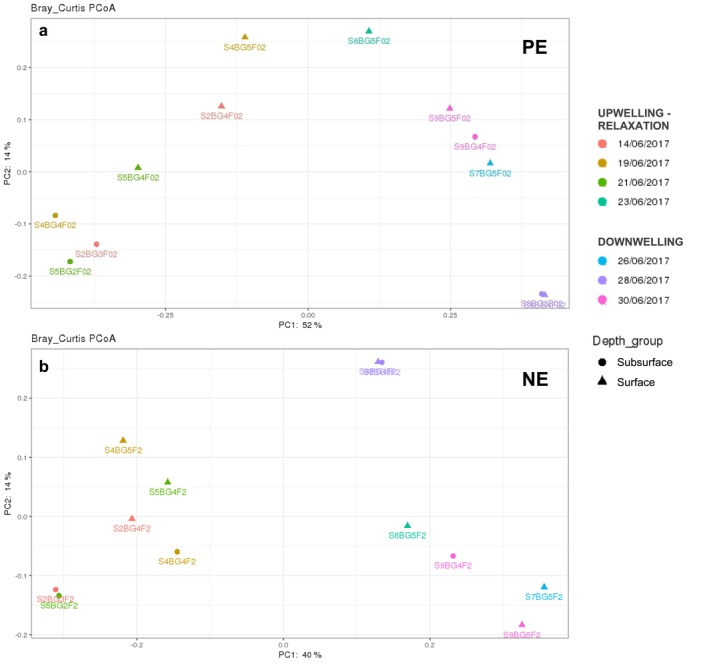
Bray Curtis principal coordinate analysis (PCoA) plots showing different sample clustering over time and depth for (a) picoeukaryotes (PE) and (b) nanoeukaryotes (NE) at BG station. Each colour represents a different sampling day and the symbol shape denotes different sampling depths: (▲) surface and (●) subsurface. F02 denote PE and F2 denote NE. The percentages of variation explained by the plotted principal coordinates (PC1 and PC2) are indicated on the axes.

To assess changes in community composition over time at BG station, beta diversity metrics were calculated for each size fraction, PE and NE. As shown in Figure 11, principal coordinate analysis (PCoA) using Bray–Curtis dissimilarities showed a community clustering by oceanographic scenario (upwelling‐relaxation vs. downwelling ‘influence’), both in PE and NE samples (Figure 11a,b, respectively). ANOSIM tests supported this view in both cases (*p* < 0.01). Pre‐downwelling samples (S2, S4 and S5) clustered separated from the downwelling group (S6, S7, S8 and S9) with the first coordinate explaining the 52% of the variance for PE and 40% for NE (Figure 11a,b). The second coordinate accounted for 14% of the variance for both size fractions. Notably, pre‐downwelling surface samples (▲) tended to cluster separately from deep ones (●), contrary to downwelling sample S8, both in NE and PE, which nearly overlapped, indicating a very similar community composition across the water column at that time.

**FIGURE 12:**
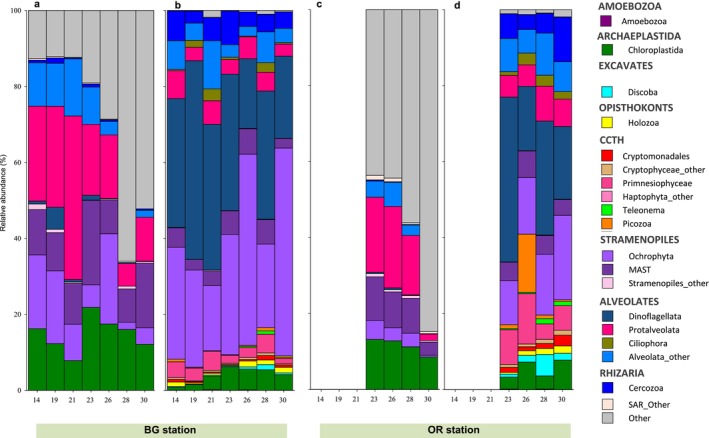
Bar plots showing the evolution in the relative abundance (%) of the major taxonomic groups of (a) picoeukaryotes and (b) nanoeukaryotes in surface waters at BG station and (c) picoeukaryotes and (d) nanoeukaryotes in surface waters at OR station at Supergroup and Subphyla level.

**FIGURE 13:**
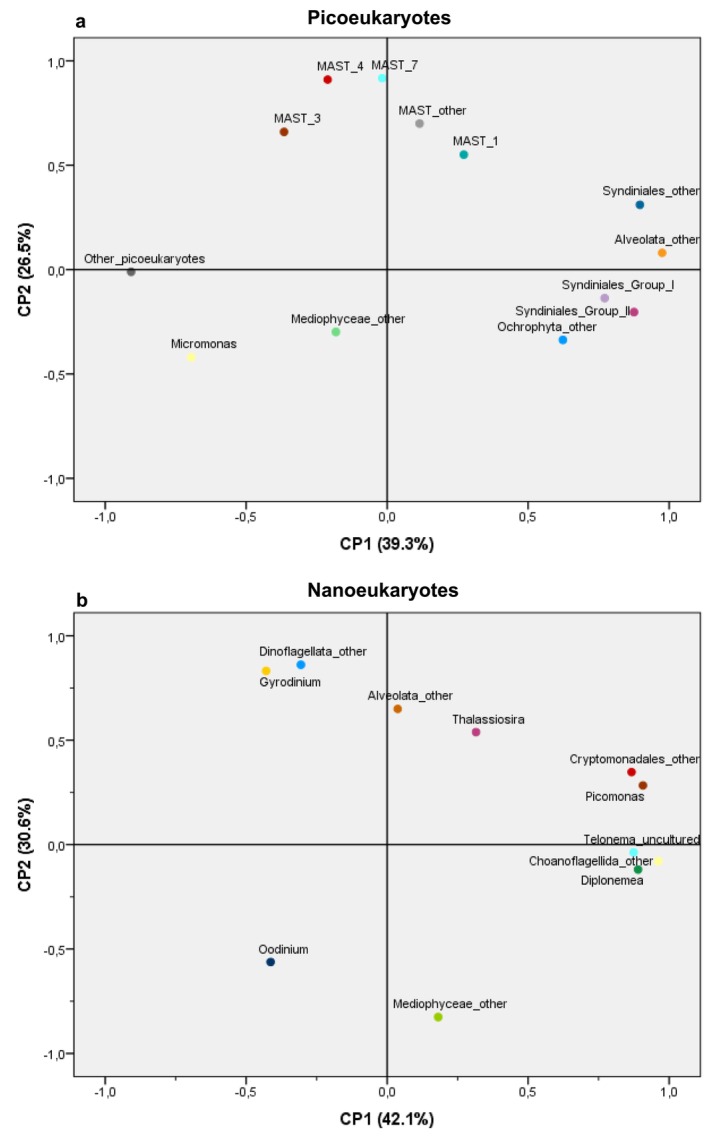
Principal component analysis (PCA) plots showing the variation in the relative abundance of the most relevant OTUs at genus level of picoeukaryotes (a) and nanoeukaryotes (b) in surface waters at BG station. The first two principal components, PC1 and PC2, were plotted.

In detail, the taxonomic composition of the small eukaryotes changed over time following the upwelling–relaxation–upwelling–downwelling–upwelling observed cycle. Within PE, an abrupt change in taxonomic composition occurred (Figure 12a). Initially, SAR supergroup dominated (> 70%) associated with upwelling, but reached a low point during downwelling (Table S3). Conversely, ‘other picoeukaryotes’ marked a peak precisely on June 28 during downwelling (66%). At higher taxonomic resolution, Protalveolata showed the greatest abundance, representing more than 40% of reads during the relaxation on June 21. It was also noted that upwelling favoured Ochrophyta, whereas MAST were promoted by relaxation. Instead, they all declined during downwelling coinciding with the increase in ‘other picoeukaryotes’ (Figure 12a). At genus level, the most relevant PE also showed significant changes in their relative abundances over time (Figure S4a–g). This variability was captured by the principal component analysis (PCA) with PC1 and PC2 explaining the 39.3% and 26.5% of the variance, respectively (Figure 13a). MAST members, which increased under relaxation (Figure S4d), were positively correlated with PC2, forming a separate cluster at the top. Meanwhile, the PC1 has positive scores for *Syndiniales*, Ocrhophyta_other and Alveolate_other, and negative for *Micromonas* and ‘other picoeukaryotes’, placed at the maximum distance from the previous ones (Figure 13a), indicating that opposite oceanographic scenarios may be involved in the success of these groups. Moreover, the SIMPER analysis indicated that ‘other picoeukaryotes’ was the primary contributor (39%) to the observed dissimilarities among hydrographic scenarios (Table S5).

Within NE community, the relative abundance of SAR supergroup hardly changed over time (Figure 12b and Table S4) unlike some of its components that exhibited large temporal variability linked to short‐term changes in hydrographic conditions. In particular, Dinoflagellata and Ochrophyta showed the opposite trend during the study period, so that the former was more abundant during relaxation and downwelling while the later remarkably increased just before and after downwelling (Figure 12b). Interestingly, a close examination of ‘minority taxa’ (< 4%), most of them belonging to the supergroups CCTH (like Picozoa, Telonema and Prymnesiophyceae) and Excavates (Discoba) revealed that their presence was remarkably higher during downwelling (Figures 12b and S4n). Also, the PCA scatter plot (Figure 13b) group these minority taxa together and far from ‘Dinoflagellate_other’ and *Gyrodimium* that thrived after upwelling (Figure S4h,i). The dinoflagellate genus ‘*Oodinium*’ increased during relaxation (Figure S4k) and was separated with negatives scores on PC1 and PC2. Also, Mediophyceae_other that showed a notable increased before and after the strong downwelling event (Figure S4l), was found insolated in the PCA diagram with negative scores on PC2 (Figure 13b). The SIMPER analysis further revealed that this taxon was the most important contributor (43%) to the differences among hydrographic scenarios (Table S5).

Subsurface samples (Figures S5a,b and S6) repeated the surface pattern (Figures 12a,b, and S4) showing similar or coincident peaks of abundance, without noticing any major difference between them (*p* > 0.05). In turn, the small eukaryotic plankton dynamics at OR station over the last week of sampling (Figure 12c,d) was significantly different (*p* < 0.05) from that at BG station (Figure 12a,b). What is remarkable about PE is the progressive increase of the unknown category ‘other picoeukaryotes’ until reached a maximum on June 30 (Figures 12c and S7g), just 2 days after peaked at BG station (Figure S4g). Instead, nanoeukaryotic minority taxa at OR (Figure 12d), and in particular *Picomonas* genus, reached a maximum at the beginning of the downwelling on June 26 (Figure S7n), in this case 2 days before they peaked at BG station (Figure S4g), to decline afterward.

## 4 Discussion

### 4.1 Taxonomic Composition of PE and NE in the Ría de Vigo During the Upwelling Season

Assessing the diversity of small eukaryotes has always been a challenging task, primarily due to the difficulties in discerning nearly imperceptible morphological differences using traditional methods such as microscopy. Molecular techniques based on rDNA genes extracted from natural assemblages emerged as a valuable tool for gaining new insights into the diversity of marine microbial plankton (Ríos‐Castro et al. 2021). Our strategy was to analyse the picoeukaryotes and nanoeukaryotes separately by means of metabarcoding, setting the size limit at 2 μm, which resulted in a significant differentiation between PE and NE assemblage's composition (*p* < 0.01, ANOSIM test). Moreover, our alpha diversity analysis revealed a considerably greater richness and diversity in NE compared with PE (Figure 8). Therefore, aligning with prior studies (De Vargas et al. 2015; Obiol et al. 2020), we suggest treating PE and NE as independent categories, to explore their unique characteristics. In this manner, our study offers a detailed description of the diversity and taxonomic composition of PE and NE in the Ría de Vigo during the upwelling season, revealing noteworthy differences between these two plankton groups.

At the broadest taxonomic level, the SAR supergroup (Stramenopiles, Alveolates and Rhizaria) gathered the vast majority of taxa identified as PE and NE in surface waters of the Ría de Vigo. These findings are consistent with prior research highlighting the prevalence of SAR lineages in marine environments (De Vargas et al. 2015; Elferink et al. 2017) and also align with the observations made by Ríos‐Castro et al. (2021) in their pioneering use of metabarcoding to assess eukaryotic diversity in the ria. The SAR clade is known for its extensive diversity (del Campo et al. 2014), displaying a wide range of morphologies and livelihoods that make them successful from the coast to the ocean (Grattepanche et al. 2018). Our study also found this rich phylogenetic diversity, which likely reflects the multiple trophic niches and functional roles of SAR members in the Ría de Vigo.

In a more detailed analysis of the taxonomic composition of PE, *Syndiniales* and MAST emerged as the primary components identified (Figure 9a). *Syndiniales*, also known as MALV (Marine Alveolates), ranked first, accounting for 21% of the relative read abundance in surface waters of the Ría de Vigo. This group belongs to a parasitic order within Protalveolata that has been documented to represent more than half of the abundance in small heterotrophic plankton samples collected during the global TARA expedition (De Vargas et al. 2015). MALV is also known to dominate a wide range of marine environments (Massana and Pedrós‐Alió 2008; Worden and Not 2008; Guillou et al. 2008; Pernice et al. 2016). However, their relative abundance is highly variable across locations, likely influenced not only by the different environmental conditions but also by the availability of hosts, given their parasitic lifestyle (Sehein et al. 2022). Next, we found MAST members that ranked second. They are believed to play a significant role as grazers of bacteria and picophytoplankton (Massana et al. 2006; Piwosz and Pernthaler 2010), and are common components of coastal and open ocean environments, mostly MAST 1, 3, 4 and 7 (Massana et al. 2004), which were also highly abundant in our study (Figure S4d). The photosynthetic genus *Micromonas* emerged as the third most abundant. These tiny organisms are known to be widely distributed in the ocean (Thomsen and Buck 1998) and especially significant in temperate coastal waters (Romari and Vaulot 2004; Not et al. 2004; Simon et al. 2017). Certainly, there is evidence that *Micromonas* was a notable taxon in shelf waters off the Ría de Vigo. However, at this location, another Mamiellophyceae (*Ostreoccocus*) clearly dominated over *Micromonas* throughout the year (Hernández‐Ruiz et al. 2018). Furthermore, it is important to note that Mamiellophyceae group clearly dominates the community of small eukaryotes in shelf waters, surpassing in abundance other heterotrophic PE like MALV. By contrast, the prominent role of non‐pigmented PE within the Ría de Vigo suggests that they could play a significant role in shaping the pigmented PE community and the microbial loop of the ria, given the complex interactions exhibited by *Syndiniales* and MAST in various marine environments (Clarke et al. 2019; Latorre et al. 2021; Lin et al. 2022; Kang and Kang 2023). However, caution is still needed when assessing the balance between pigmented and non‐pigmented PE using metabarcoding data, as the large rDNA copy number of MALV could introduce bias (Not et al. 2009), so further research is required to achieve a more comprehensive understanding of PE composition. Precisely, in our quest for knowledge about PE taxonomic composition, we realised the importance of including taxa unassigned to any known clade into the analysis. Remarkably, a large proportion of PE diversity in the Ría de Vigo grouped into this unassigned category of PE ‘other picoeukaryotes’. Unfortunately, the vast majority of metabarcoding studies to date focused only on known eukaryotic taxa or explored just a small number of groups in great detail. Only a few authors have highlighted the large share of small eukaryotic taxa yet undescribed or not existing in the reference databases (De Vargas et al. 2015; Bakker et al. 2019). This virtually hidden diversity could represent novel species and functional traits that deserve further investigation, including culture‐based methods. Further research focus on this cryptic diversity is necessary to develop our understanding and account their role in ecosystem functioning.

Metabarcoding results also revealed that NE size fraction was clearly dominated by nano‐sized diatoms and dinoflagellates. Small centric diatoms were of outstanding importance, particularly the unclassified ‘Mediophyceae_other’ (Figure 9b). This finding supports the suspicion that nano‐sized diatoms could be very important in some coastal and ocean areas (Leblanc et al. 2018). Even more important were dinoflagellates, mostly unassigned. These findings match previous studies that have separately addressed the nanoeukaryotic community, identifying Alveolates and, more specifically, the class Dinophyceae as the dominant group in various marine ecosystems (Elferink et al. 2017; Wang et al. 2021) but, just as in the case of MALV within PE community, it is necessary to take into consideration potential biases arising from the elevated number or rDNA copies of Alveolates in general (Massana et al. 2015). Furthermore, higher abundances of nano‐sized diatoms and dinoflagellates seem to be restricted to the nutrient‐rich waters of the Ría de Vigo interior, given their lack of prevalence in the nearby shelf (Hernández‐Ruiz et al. 2018), and many of the sequences recovered do not match known species, suggesting that novel minute diatoms and dinoflagellates may be present in the ria. In accordance with previous studies (Figueiras, Labarta, and Fernández‐Reiriz 2002; Froján et al. 2018), our results underline the crucial role of the Rías Baixas by amplifying the upwelling and the availability of nutrients within its borders, with diatoms and dinoflagellates of all sizes, also small ones, raising the levels of production and autotrophy of these upwelling bays.

A further observation of this study is that changes in the taxonomic composition of small eukaryotes in the Ría de Vigo were associated with differences in depth. Subsurface samples exhibited a higher degree of heterotrophy, influenced by the increase of abundance of some specific groups, such as MAST and *Syndiniales* in the case of PE or the decrease in the abundance of small centric diatoms in the case of NE (Figures 9a,b and S3). This was also aligned with biomass observations of the first part of the study (Table S2). Heterotrophy increasing with greater depths has been previously documented (Hernández‐Ruiz et al. 2018; Wu, Huang, and Zhong 2014), with temperature, light, nutrients availability and trophic interactions recognised as key factors influencing microbial plankton distribution (Morán et al. 2010; Barton et al. 2015). In this regard, the different levels of heterotrophy in our study may be reflecting a niche adaptation of PE and NE, and this effect is evident even when comparing populations separated just 10 m apart.

Additionally, we also noted that the outermost location of the Ría de Vigo displayed higher levels of unassigned PE (‘other picoeukaryotes’) and some minority NE taxa (*Picomonas* and Diplonemea) compared to the inner ria (Figure 9). This pool of diversity underexplored could hide a coastal‐ocean gradient in the pico–nanoeukaryotic assemblages similar to that observed in prokaryotes and microplankton composition (Baltar et al. 2007; Lorenzo et al. 2005). However, it seems that we are still far from capturing the full diversity of PE. Indeed, our analysis granted greater diversity to NE, but if the cryptic diversity of ‘other picoeukaryotes’ were resolved, the picture might be different. For this reason, a greater effort should be made to identify new groups to work with all the diversity that allow a more accurate vision of the ecology of small eukaryotes.

### 4.2 Short‐Term Variability of PE and NE in the Ría de Vigo

The Ría de Vigo is a highly dynamic upwelling bay, which means that superimposed on seasonal patterns, there is significant short‐term variability in hydrographic conditions (Figueiras, Labarta, and Fernández‐Reiriz 2002; Barton et al. 2015). Our sampling program captured the rapid response of microbial plankton to ever‐changing environmental conditions (Figures 2 and 3). This dynamic environment resulted in size‐structural changes within the microbial plankton community. Microplankton was clearly dominant during upwelling events, while pico‐ and nanoplankton gained importance during the downwelling episode, as revealed by chlorophyll *a* concentration (Figure 4 and Table 1) and CF rates (Figure 5 and Table 1). Such pattern has previously been documented in the Ría de Vigo (Figueiras, Labarta, and Fernández‐Reiriz 2002; Figueiras et al. 2020; Froján et al. 2014) and was also observed on the adjacent shelf (Espinoza‐González et al. 2012). Over the years, microplankton populations (e.g., diatoms and dinoflagellates) have been extensively monitored, leading to a substantial understanding of the key drivers underlying their dynamics. Instead, the small plankton dynamics remains largely underexplored. This was the context in which we decided to focus our efforts on unlocking the ‘black box’ of small eukaryotic plankton diversity, exploring which factors may be influencing their abundance and taxonomic composition.

The first thing that caught our attention when analysing the data were the simultaneous changes in the biomass and taxonomic composition of PE and NE when responding to changes in hydrographic forcing. These shifts occur on very short timescales, highlighting the immediacy of the response of small eukaryotes to short‐term changes in environmental conditions. Specifically, β‐diversity analysis supports this idea, with moderate values for both PE (40%) and NE (54%), detecting a shift in the community composition of PE and NE during the course of the study (Figure 11), likely driven by the joint action of hydrodynamic forces and biotic interactions.

Going into more detail, biomass observations clearly detected a peak of PE biomass inside the Ría de Vigo on June 28 during downwelling (Figure 6), whereas metabarcoding revealed that the vast majority of these PE were organisms still unknown in the databases (Figure 12a). Moreover, consistent with biomass results, metabarcoding also recorded a peak of ‘other picoeukaryotes’ at OR 2 days after peaked inside the ria (Figure 12c), demonstrating a strong alignment between the classical and molecular methods in our study. Interestingly, both methods detected spatial and temporal changes in the biomass and composition of the PE in the Ría de Vigo that could derive from advection processes. Thus, given that the outer zone of the ria had the highest ratio of ‘other picoeukaryotes’ and it is likely that they entered the ria during downwelling, forced by negative circulation, and exited again once positive circulation was re‐established. This assentation is supported by the PCA analysis (Table S6), that clearly shows that variability in the group ‘other picoeukaryotes’ is primary linked to the explanatory variables nitrate and velocity, indicating that advection was the primary driver of variability observed in PE.

Concerning NE, traditional methods equally recorded an increase in biomass within the Ría de Vigo during downwelling, and metabarcoding revealed that this occurred simultaneously with a change in the community composition. Most notably, there was a higher abundance of several members of CCTH and Excavates supergroups within the ria during downwelling (Figure 12b), likely dragged from OR station (Figures S4n and S7n) due to de advection of oceanic surface waters toward the coast. However, these organisms did not exit the ria once positive circulation was restored (Figure 12d), which is consistent with biomass observations (Figure 6c,d), therefore, behaving differently to their smaller counterparts. In this way, metabarcoding results would be indicating that local grazing within the ria is important enough to alter NE dynamics, but has a minimal effect over PE. Therefore, as earlier authors argued, not only physical and/or chemical features, such temperature, salinity and nutrient concentrations, influence microbial plankton dynamics. Marine microbial community also change over multiple timescales in response to different biological forces, for example predator–prey interactions, competition and viral infections that may drive strong changes in the taxonomic composition (Fuhrman, Cram, and Needham 2015). In the context of the Ría de Vigo, NE represent an important food source for higher‐level microbial predators, especially for members of microzooplankton, such as ciliates and dinoflagellates (Teixeira et al. 2011). These organisms have a certain buoyancy that would allow them to remain in the superficial layer of the water column during downwelling events (Figueiras et al. 1995), so they could be potential consumers of the NE that reach the interior of the Ría de Vigo. Unfortunately, we did not have microzooplankton data during this study. On the other hand, recent studies in the Ría de Vigo have shown evidence that NE are part of the mussels' diet, contrary to PE, which would be inefficiently captured due to its small size by adult mussels (Froján et al. 2014). In this regard, our observations suggest that the impact of mussel faming in the Ría de Vigo (67,000 tons in 2017) is significant enough to alter the composition of the small eukaryotic plankton community, specifically that of nanoplankton.

The significance of PE and NE in coastal upwelling systems often goes unnoticed, possibly due to the traditional perspective that large diatoms dominate in these areas with high nutrient loads (Kudela et al. 2005). Indeed, the prevalence of short food chains in upwelling systems is now recognised as transitional (Legendre and Rassoulzadegan 1995). Most of the time the classical food chain coexists with the microbial food, in which the biomass of organisms from smaller fractions of plankton is transferred to higher trophic levels through multiple links. The sporadic dominance of the smallest organisms and the importance of microbial loop has been previously recognised in our upwelling system (Figueiras, Labarta, and Fernández‐Reiriz 2002; Teixeira et al. 2011), which can be inferred in this study through the aforementioned short‐term variability in the pico‐ and nanoeukaryotic biomass.

Understanding the response of microbial planktonic communities to environmental conditions is crucial to predict how they will respond in a future scenario of global change. In this context, Bakun (1990) postulated that the increase in the greenhouse effect would lead to a general reinforcement of favourable upwelling winds in the main upwelling systems. However, there has been no agreement on this trend in the Iberian upwelling system, where upwelling appears to be declining, with slight changes in phytoplankton populations and an increase in toxic phytoplankton events (Álvarez‐Salgado et al. 2008; Pérez et al. 2010). However, more recent works did not find significant long‐term trends in upwelling (Otero et al. 2023), or rather the decrease–increase in favourable upwelling winds is the enhanced stratification of the water column, caused by the increase in temperature in the surface layers, which prevents the upwelled water to reach the photic layer (Sousa et al. 2020). Faced with such a scenario, with strengthened stratification, a profound change in the structure of planktonic communities can be expected with a predominance of smaller organisms and the microbial trophic chain, which could affect the high productivity of the system.

## 5 Concluding Remarks

Our study revealed that metabarcoding complements and extends the results obtained by traditional methods. We have conducted a comprehensive exploration of the composition of the pico‐ and nanoeukaryotic community and its dynamics, allowing us to observe the rapid response of these organisms to environmental changes and anticipate potential future developments. These findings are crucial for addressing fundamental issues, such as productivity in coastal upwelling systems in the context of global change. It is important to note that this study represents a first step toward understanding these populations, based on a limited number of samples. Still, it has already revealed valuable insights into their dynamics and diversity, including a significant hidden diversity. All these findings urge the need to continue researching in order to draw definitive conclusions and to gain a more complete and accurate understanding of the microbial plankton community in coastal upwelling systems. Ultimately, this work highlight the importance of combining traditional methods with molecular approaches to achieve a deeper understanding of microbial eukaryotes and ecological processes in marine environments.

## Author Contributions

**María Froján:** writing – original draft, writing – review and editing, methodology, data curation, investigation. **Marta Muñoz‐Colmenero:** data curation, software, writing – review and editing. **Isabel G. Teixeira:** writing – review and editing, methodology. **Belén Arbones:** methodology, writing – review and editing. **Carmen G. Sotelo:** methodology, writing – review and editing. **Begoña Correa:** visualization, methodology. **Francisco G. Figueiras:** writing – review and editing, conceptualization, supervision, investigation. **Carmen G. Castro:** conceptualization, supervision, writing – review and editing, investigation, funding acquisition, project administration.

## Conflicts of Interest

The authors declare no conflicts of interest.

## Supporting information


**Table S1.** Sampling strategy for Dapi (biomass) and molecular analysis (taxonomic composition) of pico‐ and nanoplankton at BG and OR stations. Sampling dates with data for both variables are shaded.
**Table S2.** Autotrophic and heterotrophic pico‐ and nanoeukaryotic plankton biomass (μg C L^−1^) at BG (surface and subsurface) and OR (surface) stations, together with the percentage corresponding to each group (in parenthesis). Total PE (total biomass of picoeukaryotes), Total NE (total biomass of nanoeukaryotes), APE (biomass of autotrophic picoeukaryotes), HPE (biomass of heterotrophic picoeukaryotes), ANE (biomass of autotrophic nanoeukaryotes) and HNE (biomass of heterotrophic nanoeukaryotes). At the bottom of the table, average ± SD for the entire data set. Shaded data correspond to the last 4 sampling days plotted in Figure 6, used to test BG and OR dynamics.
**Table S3.** Evolution in the relative abundance of the main picoplankton supergroups (%) at BG station at surface. At the bottom of the table, average ± SD for the entire period (June 2017).
**Table S4.** Evolution in the relative abundance of the main nanoplankton supergroups (%) at BG station at surface. At the bottom of the table, average ± SD for the entire period (June 2017).
**Table S5.** Summary of SIMPER test results identifying the taxa that contributed most to assemblage differences between upwelling and downwelling, based on the Bray–Curtis dissimilarity matrix.
**Table S6.** Results of the Principal Component Analysis (PCA) showing the loadings of the most relevant taxa of picoeukaryotes and nanoeukaryotes in surface waters at BG station, along with the loadings of the following explanatory variables: velocity, upwelling index, temperature and nitrate.
**Figure S1.** (a) Syn (*Synechococcus* biomass) versus APE (autotrophic picoeukaryotes biomass), (b) Relative contribution of Syn and APE (%) to total autotrophic picoplankton biomass.
**Figure S2.** Overview of the taxonomic composition of (a) picoeukaryotes and (c) nanoeukaryotes at supergroup level detected in subsurface waters at BG station. Relative abundance (%) of the different components of Stramenopiles and Alveolates belonging to (b) picoeukaryotes and (d) nanoeukaryotes.
**Figure S3.** Top 10 most abundant taxa of: (a) picoeukaryotes and (b) nanoeukaryotes in subsurface waters at BG station. Legends bellow report the different supergroup affiliation.
**Figure S4.** Temporal evolution of the most relevant OTUs at genus level in surface waters at BG station: Picoeukaryotes: (a) Ochrophyta_other, (b) Alveolata_other, (c) Syndiniales group, (d) MAST group, (e) Mediophyceae_other, (f) *Micromonas* and (g) other picoeukaryotes. Nanoeukaryotes: (h) Dinoflagellata_other, (i) *Gyrodinium*, (j) Alveolata_other, (k) *Oodinium*, (l) Mediophyceae_other, (m) *Thalassiosira*, (n) Minority taxa (*Diplonemea*, Cryptomonadales_other, Choanoflagellida_other, *Picomonas*, Telonema_uncultured). The day of the greatest abundance is highlighted in blue on the *x*‐axis. Note the different vertical scale in “g,” “l,” and “n” plots.
**Figure S5.** Bar plots showing the evolution in the relative abundance (%) of the major taxonomic groups of (a) picoeukaryotes and (b) nanoeukaryotes in subsurface waters at BG station at Supergroup and Subphyla level. No data available on June 23 and 26.
**Figure S6.** Temporal evolution of the most relevant OTUs at genus level in subsurface waters at BG station: Picoeukaryotes: (a) Ochrophyta_other, (b) Alveolata_other, (c) Syndiniales group, (d) MAST groups, (e) Mediophyceae_other, (f) *Micromonas*, and (g) other picoeukaryotes. Nanoeukaryotes: (h) *Thalassiosira*, (i) Alveolata_other, (j) Dinoflagellata_other, (k) *Gyrodinium*, (l) Mediophyceae_other, (m) Oodinium, (n) Minority taxa (*Diplonemea*, Cryptomonadales_other, Choanoflagellida_other, *Picomonas*, Telonema_uncultured). The day of the greatest abundance is highlighted in blue on the *x*‐axis. The day of the greatest abundance in surface waters at BG station is highlighted with a yellow square. Note the different vertical scale in “d,” “g,” “l,” and “n” plots.
**Figure S7.** Temporal evolution of the most relevant OTUs at genus level in surface waters at OR station: Picoeukaryotes: (a) Ochrophyta_other, (b) Alveolata_other, (c) Syndiniales group, (d) MAST groups, (e) Ochrophyta_other, (f) *Micromonas* and (g) other picoeukaryotes. Nanoeukaryotes: (h) Dinoflagellata_other, (i) *Gyrodinium*, (j) Alveolata_other, (k) Ochrophyta_other, (l) *Skeletonema*. (m) *Protaspis*, (n) Minority taxa (*Diplonemea*, Cryptomonadales_other, Choanoflagellida_other, *Picomonas*, Telonema_uncultured). The day of the greatest abundance is highlighted in blue on the *x*‐axis. The day of the greatest abundance in surface waters at BG station is highlighted with a yellow square. Note the different vertical scale in “g” plot.

## Data Availability

Data in preparation. They will be available openly on the CSIC website once the manuscript is accepted.
