# Supplementary material for: Unveiling Short‐Scale Responses: How Pico‐ and Nanoeukaryotic Plankton Navigate Environmental Variability in a Coastal Upwelling System
Source: Environ Microbiol Rep. 2025 Apr 16;17(2):e70070. doi: 10.1111/1758-2229.70070 (PMC12001073; doi:10.1111/1758-2229.70070)
Supplement: Supplementary file 1 — Table S1. Sampling strategy for Dapi (biomass) and molecular analysis (taxonomic composition) of pico‐ and nanoplankton at BG and OR stations. Sampling dates with data for both variables are shaded. Table S2. Autotrophic and heterotrophic pico‐ and nanoeukaryotic plankton biomass (μg C L−1) at BG (surface and subsurface) and OR (surface) stations, together with the percentage corresponding to each group (in parenthesis). Total PE (total biomass of picoeukaryotes), Total NE (total biomass of nanoeukaryotes), APE (biomass of autotrophic picoeukaryotes), HPE (biomass of heterotrophic picoeukaryotes), ANE (biomass of autotrophic nanoeukaryotes) and HNE (biomass of heterotrophic nanoeukaryotes). At the bottom of the table, average ± SD for the entire data set. Shaded data correspond to the last 4 sampling days plotted in Figure 6, used to test BG and OR dynamics. Table S3. Evolution in the relative abundance of the main picoplankton supergroups (%) at BG station at surface. At the bottom of the table, average ± SD for the entire period (June 2017). Table S4. Evolution in the relative abundance of the main nanoplankton supergroups (%) at BG station at surface. At the bottom of the table, average ± SD for the entire period (June 2017). Table S5. Summary of SIMPER test results identifying the taxa that contributed most to assemblage differences between upwelling and downwelling, based on the Bray–Curtis dissimilarity matrix. Table S6. Results of the Principal Component Analysis (PCA) showing the loadings of the most relevant taxa of picoeukaryotes and nanoeukaryotes in surface waters at BG station, along with the loadings of the following explanatory variables: velocity, upwelling index, temperature and nitrate. Figure S1. (a) Syn (Synechococcus biomass) versus APE (autotrophic picoeukaryotes biomass), (b) Relative contribution of Syn and APE (%) to total autotrophic picoplankton biomass. Figure S2. Overview of the taxonomic composition of (a) picoeukaryotes and (c) nanoeuk [file EMI4-17-e70070-s001.docx]

| **Site** | **Position** | **Date** | **Depth (m)** |
| --- | --- | --- | --- |
| BG station | 42º14.450’N, 8º45.6180’W | 12/06/2017 |  |
| BG station | 42º14.450’N, 8º45.6180’W | 14/06/2017 | 2, 12 |
| BG station | 42º14.450’N, 8º45.6180’W | 16/06/2017 |  |
| BG station | 42º14.450’N, 8º45.6180’W | 19/06/2017 | 2, 10.5 |
| BG station | 42º14.450’N, 8º45.6180’W | 21/06/2017 | 10.5, 26.5 |
| BG station | 42º14.450’N, 8º45.6180’W | 23/06/2017 | 2.5 |
| BG station | 42º14.450’N, 8º45.6180’W | 26/06/2017 | 2 |
| BG station | 42º14.450’N, 8º45.6180’W | 28/06/2017 | 2.5, 10 |
| BG station | 42º14.450’N, 8º45.6180’W | 30/06/2017 | 2.5, 6 |
| OR station | 42º10.2924’N, 8º52.5222’W | 12/06/2017 |  |
| OR station | 42º10.2924’N, 8º52.5222’W | 14/06/2017 |  |
| OR station | 42º10.2924’N, 8º52.5222’W | 16/06/2017 |  |
| OR station | 42º10.2924’N, 8º52.5222’W | 19/06/2017 |  |
| OR station | 42º10.2924’N, 8º52.5222’W | 21/06/2017 |  |
| OR station | 42º10.2924’N, 8º52.5222’W | 23/06/2017 | 2 |
| OR station | 42º10.2924’N, 8º52.5222’W | 26/06/2017 | 2 |
| OR station | 42º10.2924’N, 8º52.5222’W | 28/06/2017 | 10 |
| OR station | 42º10.2924’N, 8º52.5222’W | 30/06/2017 | 2 |

**Table S1**

**Table S2**

| **Station** | **Date** | **Total PE** | **Total NE** | **APE** | **HPE** | **ANE** | **HNE** |
| --- | --- | --- | --- | --- | --- | --- | --- |
| BG surface | 14/06/2017 | 27 (52) | 25(48) | 25 (93) | 2 (7) | 16 (64) | 9 (36) |
| BG surface | 19/06/2017 | 22 (51) | 21 (49) | 16 (73) | 6 (27) | 14 (67) | 7 (33) |
| BG surface | 21/06/2017 | 10 (19) | 42 (81) | 6 (60) | 4 (40) | 25 (60) | 17 (40) |
| BG surface | 23/06/2017 | 31 (53) | 27 (47) | 20 (65) | 11 (35) | 16 (59) | 11 (41) |
| BG surface | 26/06/2017 | 72 (64) | 41 (36) | 51 (71) | 21 (29) | 25 (61) | 16 (39) |
| BG surface | 28/06/2017 | 115 (50) | 115 (50) | 81 (70) | 34 (30) | 87 (76) | 28 (24) |
| BG surface | 30/06/2017 | 22 (42) | 31 (58) | 17 (77) | 5 (23) | 21 (68) | 10 (32) |
| **BG surface** | **average ± SD** | **43±37**  (47 ± 14) | **43±33**  (53 ± 14) | **27±24**  (73 ± 10) | **12±12**  (27 ± 10) | **29±26**  (65 ± 6) | **14±7**  (35 ± 6) |
| BG subsurface | 14/06/2017 | 8 (24) | 25 (76) | 6 (75) | 2 (25) | 12 (48) | 13 (52) |
| BG subsurface | 19/06/2017 | 6 (16) | 31 (84) | 4 (67) | 2 (33) | 18 (58) | 13 (42) |
| BG subsurface | 21/06/2017 | 6 (21) | 22 (79) | 2 (33) | 4 (67) | 7 (32) | 15 (68) |
| BG subsurface | 28/06/2017 | 102 (55) | 83 (45) | 79 (77) | 23 (23) | 59 (71) | 24 (29) |
| BG subsurface | 30/06/2017 | 37 (49) | 38 (51) | 33 (89) | 4 (11) | 26 (68) | 12 (32) |
| **BG subsurface** | **average ± SD** | **32±41**  (33 ± 18) | **40±25**  (67 ± 18) | **20 ± 26**  (68 ± 21) | **7 ± 9**  (32 ± 21) | **24 ± 21**  (55 ± 16) | **15 ± 5**  (45 ± 16) |
| OR surface | 23/06/2017 | 10 (42) | 14 (58) | 4 (40) | 6 (60) | 8 (57) | 6 (43) |
| OR surface | 26/06/2017 | 36 (38) | 60 (63) | 21 (58) | 15 (42) | 42 (70) | 18 (30) |
| OR surface | 28/06/2017 | 42 (47) | 48 (53) | 28 (67) | 14 (33) | 34 (71) | 14 (29) |
| OR surface | 30/06/2017 | 115 (70) | 50 (30 | 87 (76) | 28 (24) | 38 (76) | 12 (24) |
| **OR surface** | **average ± SD** | **51±45**  (49 ± 14) | **43±20**  (51±14) | **35±36**  (60 ± 15) | **15±9**  (40 ± 15) | **30±15**  (68 ± 8) | **12±5**  (32 ± 8) |

|  | **% Archaeplastida** | **% SAR** | **% Other picoeukaryotes** |
| --- | --- | --- | --- |
| 12/06/2017 |  |  |  |
| 14/06/2017 | 16.22 | 71.15 | 12.62 |
| 16/06/2017 |  |  |  |
| 19/06/2017 | 12.28 | 75.61 | 12.10 |
| 21/06/2017 | 7.84 | 79.81 | 12.34 |
| 23/06/2017 | 21.80 | 59.16 | 19.03 |
| 26/06/2017 | 17.46 | 53.94 | 28.60 |
| 28/06/2017 | 16.02 | 17.98 | 66.00 |
| 30/06/2017 | 12.12 | 35.65 | 52.23 |
| **Entire**  **period** | **15 ± 5** | **56 ± 23** | **29 ± 22** |

**Table S3**

**Table S4**

|  | **% Archaeplastida** | **% Amoebozoa** | **% Excavata** | **% Opisthokonts** | **% Hacrobia** | **% SAR** | **% Other nanoeukaryotes** |
| --- | --- | --- | --- | --- | --- | --- | --- |
| 12/06/2017 |  |  |  |  |  |  |  |
| 14/06/2017 | 0.06 | 0.70 | 0.26 | 1.17 | 6.00 | 91.72 | 0.09 |
| 16/06/2017 |  |  |  |  |  |  |  |
| 19/06/2017 | 0.04 | 1.53 | 0.09 | 0.30 | 4.13 | 93.77 | 0.15 |
| 21/06/2017 | 0.21 | 3.56 | 0.26 | 0.47 | 5.94 | 87.77 | 1.80 |
| 23/06/2017 | 0.04 | 6.14 | 0.21 | 0.22 | 2.76 | 90.50 | 0.13 |
| 26/06/2017 | 0.13 | 5.47 | 0.60 | 1.53 | 4.11 | 87.78 | 0.39 |
| 28/06/2017 | 0.02 | 5.39 | 1.36 | 1.22 | 8.50 | 82.50 | 1.00 |
| 30/06/2017 | 0.05 | 4.07 | 0.47 | 1.46 | 3.02 | 90.55 | 0.37 |
| **Entire**  **period** | **0.1 ± 0.1** | **3.8 ± 2.1** | **0.5 ± 0.4** | **0.9 ± 0.6** | **4.9 ± 2** | **89.2 ± 3.6** | **0.6 ± 0.6** |

| **PICOEUKARYOTES**  **Taxon** | **Average Dissimilarity** | **Contribution (%)** | **Cumulative (%)** |
| --- | --- | --- | --- |
| ‘other picoeukaryotes’ | 15.03 | 39.21 | 39.21 |
| Mediophyceae_other | 6.35 | 16.56 | 55.77 |
| Ochrophyta_other | 4.39 | 11.46 | 67.23 |
| Alveolata_other | 3.15 | 8.21 | 75.44 |
| Syndinales Group I | 2.76 | 7.19 | 82.64 |
| Syndiniales Group II | 1.87 | 4.89 | 87.53 |
| *Micromonas* | 1.52 | 3.97 | 91.5 |
| MAST_7 | 1.23 | 3.20 | 94.7 |
| Syndiniales_other | 1.16 | 3.04 | 97.74 |
| MAST_3 | 0.87 | 2.26 | 100 |
| **NANOEUKARYOTES**  **Taxon** | **Average Dissimilarity** | **Contribution (%)** | **Cumulative (%)** |
| Mediophyceae_other | 17.34 | 43.04 | 43.04 |
| Dinoflagellata_other | 4.49 | 11.16 | 54.19 |
| *Skeletonema* | 3.23 | 8.01 | 62.2 |
| *Thalasiossira* | 2.96 | 7.34 | 69.54 |
| Alveolata_other | 2.48 | 6.15 | 75.7 |
| Ochrophyta_other | 2.46 | 6.11 | 81.8 |
| *Gyrodinium* | 2.06 | 5.11 | 86.91 |
| *Protaspis* | 1.84 | 4.28 | 91.49 |
| *Oodinium* | 1.74 | 4.33 | 95.82 |
| *Chatonella* | 1.68 | 4.18 | 100 |

**Table S5**

| **PICOEUKARYOTES**  **Explanatory variables loadings** | **PC1** | **PC2** |
| --- | --- | --- |
| VELOCITY | 0.475 | -0.321 |
| UPWELLING INDEX | -0.003 | 0.391 |
| TEMPERATURE | 0.079 | -0.347 |
| NITRATE | -0.392 | 0.185 |
| **Taxon loadings** |  |  |
| *Micromonas* | 0.081 | -0.079 |
| Syndiniales Group I | -0.153 | 0.176 |
| Syndiniales Group II | -0.165 | 0.023 |
| Syndiniales_other | -0.112 | 0.073 |
| Alveolata_other | -0.223 | 0.177 |
| MAST_1 | -0.034 | -0.057 |
| MAST_3 | -0.019 | 0.069 |
| MAST_4 | 0.005 | -0.012 |
| MAST_7 | 0.002 | 0.041 |
| MAST_other | -0.001 | -0.013 |
| Mediophyceae_other | 0.001 | -0.868 |
| Ochrophyta_other | -0.208 | 0.362 |
| ‘other picoeukaryotes’ | 0.913 | 0.173 |
| *Variance explained (%)* | 79 | 11 |
|  |  |  |

| **NANOEUKARYOTES**  **Explanatory variables loadings** | **PC1** | **PC2** |
| --- | --- | --- |
| VELOCITY | 0.486 | -0.588 |
| UPWELLING INDEX | 0.023 | 0.258 |
| TEMPERATURE | 0.305 | 0.346 |
| NITRATE | -0.331 | -0.591 |
| **Taxon loadings** |  |  |
| Mediophyceae_other | 0.941 | 0.229 |
| Dinoflagellata_other | -0.238 | 0.679 |
| Alveolata_other | -0.132 | -0.249 |
| *Gyrodinium* | -0.151 | 0.351 |
| *Oodinium* | 0.017 | -0.402 |
| *Thalasiossira* | -0.017 | 0.091 |
| *Chattonella* | -0.065 | -0.138 |
| Ochrophyta_other | -0.106 | 0.331 |
| Diplonemea | 0.003 | -0.031 |
| Cryptomonadales_other | -0.0001 | 0.005 |
| Choanoflagellida_other | 0.001 | -0.003 |
| *Picomonas* | -0.002 | -0.002 |
| Telonema_uncultured | 0.001 | -0.014 |
| *Variance explained (%)* | 88 | 6 |
|  |  |  |

**Table S6**

APE

(µmol C L^-1^)

**a**

**b**

**Figure S1**

**Nanoeukaryotes**

**Picoeukaryotes**

Amoebozoa

0.19 %

Archaeplastida

2.95 %

Rhizaria

0.85 %

SAR_other

0.16 %

Excavates

0.47 %

Other nanoeukaryotes

0.39 %

SAR_other

0.06 %

CCTH

0.01 %

Opisthokonts

1.04 %

CTTH

3.92 %

Rhizaria

9.45 %

**c**

**a**

Alveolates

42.64 %

Other picoeukaryotes

24.94 %

Stramenopiles

31.90 %

Archaeplastida

7.64 %

Alveolates

49.64 %

Stramenopiles

23.76 %

**d**

**b**

**STRAMENOPILES**

12

16

13

59

86

14

85

68

**ALVEOLATES**

40

**Figure S2**

**b**

**a**

**Nanoeukaryotes**

**Picoeukaryotes**

Relative abundance (%)

Syndiniales_group I

*Oodinium*

Ochrophyta_other

*Protaspis*

Mediophyceae_other

*Thalassiosira*

*Gyrodinium*

Alveolata_other

Dinoflagellata_other

MAST 4

MAST 7

*Micromonas*

Alveolata_other

MAST 3

Ochrophyta_other

Syndiniales Group II

Syndiniales Group I

Other picoeukaryotes

**BG** Subsuperficie

Syndiniales_other

Bacillariophyceae_other

16%

2%

8%

10%

12%

14%

6%

4%

0%

30%

20%

10%

0%

40%

50%

60%

**Figure S3**

CCTH

Rhizaria

Excavates

SAR_other

Archaeplastida

Stramenopiles

Alveolates

Other picoeukaryotes

**Picoeukaryotes**

Ochrophyta _other

**a**

Alveolata_other

Mediophyceae_other

Micromonas

Other picoeukaryotes

**b**

**c**

**d**

**e**

**f**

**g**

**Nanoeukaryotes**

**h**

Dinoflagellata_other

Gyrodinium

Alveolata_other

Oodinium

Mediophyceae_other

Thalasiossira

**i**

**j**

**k**

**l**

**m**

**n**

**Figure S4**

**b**

**a**

**RHIZARIA**

**ALVEOLATES**

**STRAMENOPILES**

**CCTH**

**OPISTHOKONTS**

**EXCAVATES**

**ARCHAEPLASTIDA**

**AMOEBOZOA**

**Figure S5**

**Picoeukaryotes**

Mediophyceae_other

Alveolata_other

Ochrophyta_other

**a**

*Micromonas*

Other picoeukaryotes

**b**

**c**

**d**

**e**

**f**

**g**

**Nanoeukaryotes**

**h**

*Thalassiosira*

Alveolata_other

Dinoflagellata_other

*Gyrodinium*

Mediophyceae_other

*Oodinium*

**i**

**j**

**k**

**l**

**m**

**n**

**Figure S6**

Ochrophyta_other

**a**

Other picoeukaryotes

Dinoflagellata_other

*Gyrodinium*

Ochrophyta_other

**b**

**c**

**d**

**e**

**f**

**g**

**h**

*Protaspis*

**i**

**j**

**k**

**l**

**m**

**n**

**Nanoeukaryotes**

**Picoeukaryotes**

Chlorophyta_other

*Micromonas*

Alveolata_other

*Skeletonema*

Alveolata_other

**Figure S7**
